# Micronutrient Deficiencies Presenting with Optic Disc Swelling Associated with or without Intracranial Hypertension: A Systematic Review

**DOI:** 10.3390/nu14153068

**Published:** 2022-07-26

**Authors:** Gavin Reynolds, Simon Epps, Alyson Huntley, Denize Atan

**Affiliations:** 1 Bristol Eye Hospital, Bristol BS1 2LX, UK; simon.epps@uhbw.nhs.uk (S.E.); denize.atan@bristol.ac.uk (D.A.); 2 Translational Health Sciences, University of Bristol, Bristol BS8 1UD, UK; 3 Centre for Academic Primary Care, University of Bristol, Bristol BS8 2PS, UK; alyson.huntley@bristol.ac.uk

**Keywords:** idiopathic intracranial hypertension (IIH), obesity, nutritional deficiency, papilledema, disc swelling

## Abstract

Idiopathic intracranial hypertension (IIH) is a neurological disorder characterised by optic disc swelling secondary to raised intracranial pressure (ICP) of unknown cause. Obesity is the most established and prevalent risk factor in developed countries. As obesogenic diets are high in calories and nutrient-poor, there may be associated nutritional deficiencies that contribute to the clinical presentation of IIH. Yet none, aside from iron deficiency, are currently included in the inclusion or exclusion criteria for the diagnosis of IIH. Our primary aim was to determine which micronutrient deficiencies, aside from iron deficiency, could present with optic disc swelling associated with or without intracranial hypertension that could potentially meet current IIH diagnostic criteria. To this end, we conducted a systematic search of articles published between 1 January 1980 and 18 December 2020 reporting cases of optic disc swelling associated with micronutrient deficiencies. In total, 65 cases met the eligibility criteria from initial searches: all were case reports and case series with a high risk of bias. Our findings suggest that patients with IIH or unexplained optic disc swelling ought to be screened, investigated, and treated for associated micronutrient deficiencies in vitamin A, B1 and B12; and weight loss interventions in IIH patients ought to promote better nutrition in addition to overall calorie restriction.

## 1. Introduction

Idiopathic intracranial hypertension (IIH), also known as pseudotumor cerebri, is a neurological disorder characterised by raised intracranial pressure (ICP) of unknown cause. IIH can lead to chronic headache and visual loss with a 1–2% annual risk of blindness [1]. The annual estimated costs of IIH in the UK exceed GBP50 million and more than USD444 million in the US because of loss of productivity of working age adults and frequent hospitalisations [2,3].

Patients with IIH have papilledema but normal neurological examinations (except for sixth cranial nerve palsies), normal cerebrospinal fluid composition and no structural cause of intracranial hypertension based on brain imaging [4]. Raised ICP is defined as lumbar puncture opening pressure ≥25 cm CSF in adults (≥28 cm CSF in children), but the diagnosis of IIH is still considered probable if the opening pressure is <25 cm CSF and other diagnostic criteria are met [4].

Although the pathogenesis of IIH is not understood, there are several established risk factors (Table 1). Obesity is the strongest modifiable risk factor as the annual incidence of IIH rises from 0.5–2 per 100,000 in the general population to 12–20 per 100,000 of obese women of reproductive age [5] and the clinical features of IIH improve in patients who lose weight [6]. Hence, the management involves changes to diet, medications and/or bariatric surgery to promote weight loss.

While it is self-evident that patients who consume fewer nutrient-poor ‘junk’ foods are more likely to lose weight, the main emphasis of IIH management is usually calorie restriction rather than better nutrition. However, micronutrient deficiencies are common among the obese, particularly vitamin B12 (cobalamin) [7], vitamin B9 (folate) [8], and iron deficiency [9], and up to 85% of obese people undergoing bariatric surgery have ≥1 pre-operative micronutrient deficiency [10]. Iron deficiency anaemia is an established risk factor for IIH (Table 1) [11]. Hence, diets that restrict calories without recognising the importance of good nutrition may actually exacerbate nutritional deficiencies and the clinical presentation of IIH.

Given the links between diet, obesity and IIH and the high prevalence of nutritional deficiencies among obese individuals, the primary aim of this systematic review was to determine which nutritional deficiencies, aside from iron deficiency, could present with optic disc swelling associated with or without intracranial hypertension that could potentially meet current IIH diagnostic criteria. The secondary aims were to determine population risk factors, causes, clinical features, investigations, interventions (including dietary), and outcomes.

## 2. Materials and Methods

### 2.1. Protocol

The results of this systematic review are reported following the Preferred Reporting Items for Systematic Reviews and Meta-Analyses (PRISMA) guidelines and checklist (Supplementary Material Figure S1) [12].

### 2.2. Search Strategy

A systematic search was conducted for articles published between 1 January 1980 and 18 December 2020 in Medline, Embase, Web of science (core collection), and Scopus; non-English manuscripts were included if an English translation was available.

The search strategy included “nutritional” keywords, e.g., “Nutritional”, “Diet”, “Vitamin”, combined with keywords associated with optic disc pathology, e.g., “Disc”, “IIH”, “optic neuropathy”, using “AND” and “OR” operators, accounting for variations in spelling. Full search criteria in Medline format are provided (Supplementary Table S1).

### 2.3. Eligibility Criteria

#### 2.3.1. Inclusion Criteria

Any reported cases of optic disc swelling associated with one or more nutritional deficiency. We included cases with presumed nutritional deficiencies (when biochemical test results were not reported) if there was supporting evidence from the clinical presentation and response to treatment. For example, many reported cases of thiamine deficiency were diagnosed based on their clinical presentation with one or more of Wernicke’s triad (ophthalmoparesis, ataxia, confusion) rather than biochemical evidence of thiamine deficiency but resolved with thiamine supplementation; all these cases had papilledema to meet the inclusion criteria of this review.

#### 2.3.2. Exclusion Criteria

Cases with co-existent morbidities known to cause optic disc swelling or IIH were excluded, e.g., ischemic optic neuropathy or Leber’s Hereditary Optic Neuropathy, even if they also had nutritional deficiencies. We also excluded cases with iron deficiency and/or severe anaemia (defined as haemoglobin < 8.0 g/dL) [13] as they are already established risk factors for IIH and have been reviewed elsewhere [11,14]. Reports of epidemic optic neuropathy, e.g., the Cuban epidemic, were not included as individual patient-level information was lacking and there is still some uncertainty about their etiology; these cases are considered in the discussion.

### 2.4. Data Extraction

Two reviewers (GR, SE) screened titles +/− abstracts that met the inclusion and exclusion criteria. Articles were imported into Rayyan and duplicates removed [15]. Disagreements between reviewers were resolved after reviewing the full text articles. As all articles were case reports or case series, the risk of bias was assessed following CARE guidelines [16].

A standardised table was used to extract data by 2 reviewers (GR, SE) on patient characteristics (age, sex, weight), cause of nutrient deficiency (e.g., dietary, anorexia, vomiting, malabsorption), clinical presentation (symptoms, examination findings including visual acuity, fields, disc appearances), investigations (neuroimaging, lumbar puncture, blood tests), interventions, and outcomes. Additional information (maternal and fetal outcome) was extracted for pregnant cases.

## 3. Results

In total, 1957 articles were screened, of which 59 publications met the eligibility criteria (Figure S1, Tables S1 and S2). All 59 articles were case reports or case series (65 cases in total) of patients with nutritional deficiencies in vitamin A (18.5%; 12/65 cases), thiamine (61.5%; 40/65 cases), vitamin B12 (16.9%; 11/65 cases), and undetermined micronutrients (3%; 2/65 cases) presenting with optic disc swelling. Most cases were female (66.1%; 43/65), with an average age of 23.4 years (range 15 months to 56 years) (Table 2 and Tables S3–S8). Poor intake (e.g., poor diet, restrictive eating, anorexia, nausea, and vomiting) or malabsorption (e.g., celiac disease, bypass surgery, cystic fibrosis) were the two main causes of nutritional deficiencies (Table 2 and Tables S3–S8). Few published cases (15.4%; 10/65) reported body mass index (BMI) and/or weight; of the cases in which BMI was reported, 71.4% (5/7) were overweight or obese as defined by BMI ≥ 25 (Tables S3–S8).

### 3.1. Vitamin A Deficiency

**Figure 1:**
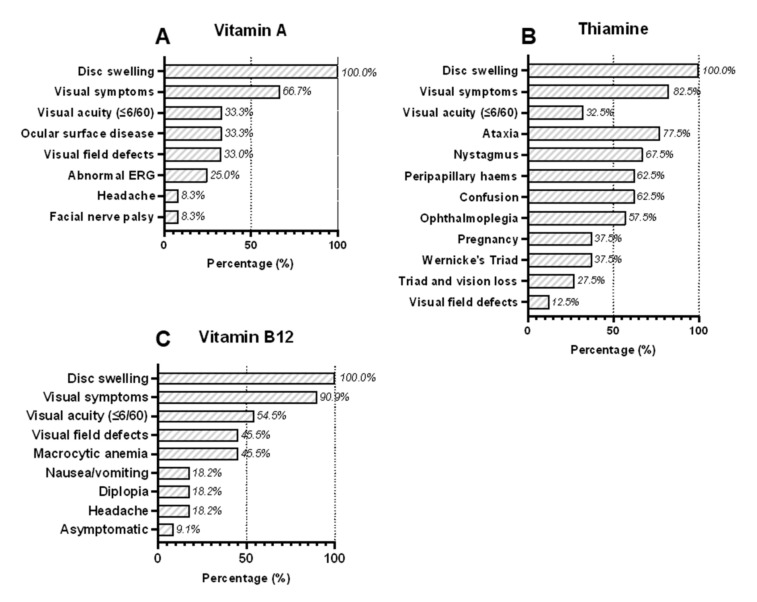
Clinical symptoms and signs of patients presenting with bilateral optic disc swelling associated with deficiencies in (**A**) vitamin A, (**B**) thiamine, and (**C**) vitamin B12.

All except one case of vitamin A deficiency were children (mean age = 10 years, range = 3–27 years) and the majority (75%; 9/12) were male (Table 2 and Tables S3 and S4) [17,18,19,20,21,22,23] The causes of vitamin A deficiency were attributed either to poor intake (66.6%; 8/12) or malabsorption (33.3%; 4/12). Clinical symptoms were reported in 9/12 cases, including visual dysfunction (66.7%; 8/12), ocular surface disease or dry eyes (33.3%; 4/12), and headache (8.3%; 1/12) (Figure 1A). Neuroimaging results were reported in 91.7% (11/12) of cases and no abnormalities were detected in the majority (81.8%, 9/11) while 70% (7/10) who had a lumbar puncture had raised opening pressure) (Table 3 and Tables S3 and S4). In those cases with abnormal neuroimaging results, the findings were ‘thickened optic nerve sheaths’ in one case [17] and ‘bony hypertrophy of the optic nerve canals’ in the other [20]. All except one case with facial nerve palsy (91.2%; 11/12) met the current diagnostic criteria for definite or probable IIH (Table 3).

All patients with vitamin A deficiency were treated with oral vitamin A supplementation and 50% (6/12) made a full recovery; 16.7% (2/12) also received acetazolamide: one recovered normal vision and the other developed optic atrophy (Table 4). Patients who had better visual acuities at presentation and shorter duration of symptoms tended to make complete recoveries, irrespective of the severity of their vitamin A deficiency. Cases with poor visual acuities at presentation were less likely to recover their visual function, despite treatment.

### 3.2. Vitamin B1 (Thiamine) Deficiency

Most cases of optic disc swelling caused by thiamine deficiency (90%; 36/40) [24,25,26,27,28,29,30,31,32,33,34,35,36,37,38,39,40,41,42,43,44,45,46,47,48,49,50,51,52,53,54,55,56,57,58,59,60,61] were young women (mean age = 28 years, range = 11–56 years), matching the typical demographic of IIH patients (Table 2 and Tables S5 and S6). The cause of thiamine deficiency was predominately due to reduced intake (62.5%; 25/40) due to anorexia, nausea, vomiting or restrictive diets. Many of these women (60%; 15/25) had developed hyperemesis gravidarum during pregnancy, which was associated with five fetal deaths and two maternal deaths. The remainder (37.5%; 15/40) developed thiamine deficiency from malabsorption. The diagnosis of thiamine deficiency was often based on the presence of one or more of Wernicke’s triad rather than biochemical evidence of thiamine deficiency, which was only reported in 32.5% (13/40) of cases (Figure 1B and Tables S5 and S6). The complete triad was observed in 37.5% (15/40) of cases.

Visual symptoms (82.5%; 31/40), reduced visual acuity ≤ 6/60 (32.5%; 13/40), and nystagmus (67.5%; 20/40) were common clinical features and 62.5% (25/40) had haemorrhagic disc swelling. Visual field results were only reported in 12.5% (5/40) cases; 4/5 had central scotomata and 1/5 case had “incongruent” field loss. Intracranial pressure was consistently within the normal range (Table 3). Ophthalmoplegia from sixth nerve palsy does not exclude the diagnosis of IIH, although ataxia and nystagmus are neurological signs that would normally exclude the diagnosis. Due to the inclusion criteria of this systematic review, all the patients we identified with thiamine deficiency had bilateral optic disc swelling; 7.5% (3/40) did not have ataxia or nystagmus and would meet the diagnostic criteria for IIH. Neuroimaging results were reported in 80% (32/40) of cases, and most (78.1%, 25/32) described T2/FLAIR changes within the periaqueductal grey, thalamus, and/or basal ganglia but no structural abnormalities (Table 3 and Tables S5 and S6). Lumbar puncture opening pressures were reported in 25.7% (11/40) of cases and were consistently normal (Table 3 and Tables S5 and S6).

Most cases (77.5%; 31/40) made a full recovery with intravenous thiamine supplementation, but 4/40 (10.0%) cases died (two from hyperemesis gravidarum, two from malabsorption) and there were five fetal deaths from 15 pregnancies (33.3%) (Table 4). In most patients (87.5%; 35/40), the response to treatment was rapid and complete. There was no obvious relationship between the response to treatment and visual acuities or duration of symptoms at presentation.

### 3.3. Vitamin B12 (Cobalamin) Deficiency

We identified 11 cases of vitamin B12 deficiency presenting with optic disc swelling, which were all caused by poor intake and none from malabsorption (Table 3 and Table S7) [62,63,64,65,66,67,68,69,70,71,72]. Due to the exclusion criteria of this review, none of these cases were severely anaemic (defined as haemoglobin ≤8.0 g/dL) [13]. Furthermore, there were no cases caused by pernicious anaemia (vitamin B12 deficiency associated with deficient intrinsic factor and autoimmune gastritis). Visual symptoms (90.9%, 10/11), nausea/vomiting (45.4%; 5/11), diplopia (18.2%; 2/11) and headache (18.2%; 2/11) were the most common symptoms and one case (9.1%; 1/11) was asymptomatic (Figure 1C). These cases were mainly young men 81.8%; 9/11) (mean age = 20.3 years, range = 15 months to 47 years). Five cases (41.7%; 5/12) had other associated micronutrient deficiencies (vitamin A, folate, and/or thiamine). Visual fields were reported in 45.5% (5/11) of cases; two had centrocaecal scotomata affecting one or both eyes (both had >1 nutritional deficiency) and the other three had enlarged blind spots or peripheral visual field loss. Neuroimaging results were reported in 90.9% (10/11) of cases and most were described as normal (80%, 8/10) (Table 3 and Table S7). Those with abnormal neuroimaging results had non-specific abnormalities that were unrelated to their diagnosis: diffuse cerebral atrophy in one case [67] and ischemic lesions in another [72]. Of four patients who had a lumbar puncture, three had elevated opening pressures ≥25 cm CSF (Table 3 and Table S7). All patients with B12 deficiency met current diagnostic criteria for definite or probable IIH.

Vitamin B12 levels were reported with their normal ranges in 81.8% (9/11) of cases but haemoglobin levels and mean corpuscular volumes (MCV) were only reported with their normal ranges in 18.2% (2/11) of cases (1 macrocytic; 1 normocytic) (Table S7). Hypersegmented neutrophils indicate early megaloblastic anaemia [73], and were reported in 18.2% (2/11) of patients. Overall, there was no obvious correlation between vitamin B12 and haemoglobin values. Finally, most cases (63.6%; 7/11) recovered with supplements and one was treated with acetazolamide (Table 4).

### 3.4. Presumed Nutritional Deficiencies

There were two reported patients who presented with optic disc swelling, headache and raised opening pressure on lumbar puncture due to presumed nutritional deficiencies who met IIH diagnostic criteria (Table 2, Table 3 and Table S8) [74,75]. Both cases were young women (mean = 14 years) with celiac disease whose symptoms completely resolved on gluten-free diets and acetazolamide (Table 4). Both cases had normal neuroimaging results and raised opening pressure on lumbar puncture (Table 3 and Table S8).

Numbers refer to the percentage of patients with each reported clinical symptom and sign associated with deficiencies in (A) vitamin A (12 cases), (B) thiamine (40 cases), and (C) vitamin B12 (11 cases). If a particular symptom or sign was not reported, then we assumed it was not present. Wernicke’s triad is defined as ophthalmoplegia, altered mental status (confusion) and ataxia.

### 3.5. Risk of Bias Assessment

Overall, compliance with the 13 standards set out by CARE guidelines for reporting case reports/series [16] was good, except for the recording of patient consent, perspective and clinical timeline (Figure S2). Weight, BMI, normal ranges for biochemical test results were infrequently reported. There was a significant bias in vision reporting, dependent on journal specialty; visual acuity was reported in 14/15 (93%) ophthalmology, 5/12 (42%) neurology, and 18/32 (56%) ‘other’ specialty journals (e.g., paediatric, medical, obstetric) (Table S9). In total, 37/59 (63%) documented vision. These biases in reporting were statistically significant (Chi-square = 8.157; *p*-value = 0.017).

## 4. Discussion

We found 65 cases of optic disc swelling associated with deficiencies in vitamins A, B1 and B12; many (41.5%; 27/65) of these cases met current diagnostic criteria for IIH, particularly those with vitamin A or B12 deficiency (Table 3). As few published cases reported BMI and/or weight, our interpretation of this data is limited; however, 71.4% (5/7) of cases in which BMI was reported were overweight or obese (Tables S3–S8). Poor intake or malabsorption were the two main causes of micronutrient deficiency in specific patient groups: vitamin A deficiency in children; thiamine deficiency in women of reproductive age; and vitamin B12 deficiency in men. In part, these age and sex differences reflect the differing nutritional demands and prevalence of micronutrient deficiencies among men, women and children in general: globally, children are most at risk of vitamin A deficiency because they have higher vitamin A requirements [76]; thiamine requirements increase during pregnancy and lactation [77], and vitamin B12 deficiency is more prevalent among men [78]. Hence, there may be physiological factors underlying these interactions between age, sex and nutrition.

Although we used robust systematic review methodology and assessment tools in this study, it is possible that some of the cases we identified had optic disc swelling and their nutritional deficiencies were coincidental. However, this hypothesis does not explain why 65% (42/65), recovered completely with nutritional supplementation alone (Table 4). The number of cases reported here are relatively small, but as deficiencies in vitamin A, B1 and B12 do not currently feature in the diagnostic or exclusion criteria for IIH or cases with unexplained optic disc swelling, the tests are often difficult to arrange and not universally available, and as awareness of the importance of nutritional deficiencies among clinicians is generally low, it is likely that many cases of optic disc swelling associated with micronutrient deficiencies are currently missed [79].

Although hypervitaminosis A is a well-established risk factor for intracranial hypertension [80], our results suggest that too little vitamin A is also a risk. Ocular surface and retinal dysfunction are well-established signs of vitamin A deficiency but were not universally present in the cases reported here (Figure 1A and Tables S3 and S4). Instead, reduced visual acuity, peripheral visual loss and headache as well as raised lumbar puncture opening pressure were reported problems. Neuroimaging results were predominately normal: one case had enlarged optic nerve sheaths consistent with optic disc swelling, whereas the other had bony hypertrophy of the optic canals, which has been described previously in another case of vitamin A deficiency [81]. Early treatment with vitamin supplements, rather than carbonic anhydrase inhibitors, led to rapid improvement in most cases. Importantly, vitamin A deficiency can result from inadequate intake or malabsorption, e.g., celiac disease, and so patients with unexplained optic disc swelling ought to be investigated for these possibilities.

We found that cases of optic disc swelling from thiamine deficiency were mostly women of reproductive age, matching the typical demographic of IIH patients. Inadequate intake was the commonest cause, particularly in pregnant women with hyperemesis gravidarum. Although Wernicke’s triad does not include optic disc swelling, a young woman who developed haemorrhagic optic disc swelling following prolonged vomiting was described in Wernicke’s original publication in 1881 [82]. Indeed, optic disc swelling was detected in 4% of people with Wernicke–Korsakoff syndrome (WKS) in a Singapore prisoner-of-war hospital [83]. Thiamine is found in some fresh fruits (bananas, oranges), peas, nuts, wholegrain bread, fortified cereals and liver. Since endogenous stores last only 1–3 weeks, thiamine deficiency can occur rapidly and acutely [84]. Thiamine deficiency is well described among those who consume excess alcohol and little else, because alcohol is high in calories but nutrient poor. In non-alcoholic cases of WKS, there is evidence that higher body weight protects against eye movement problems [85]. Moreover, bariatric surgery is an additional risk factor for WKS secondary to malabsorption in obese individuals. Thiamine deficiency is often diagnosed clinically, without biochemical confirmation, because thiamine compounds are photosensitive and only stable for a few hours at room temperature, so specimens need to be transported on ice and in darkness, then rapidly frozen for storage [86]. As awareness of non-alcoholic WKS among clinicians is generally low and thiamine tests are difficult to arrange, many cases remain undetected and untreated until they start to develop one or more of Wernicke’s triad [85]. Hence, milder cases of thiamine deficiency may present with isolated optic disc swelling that remains undiagnosed until additional neurological disease develops. These issues create clear detection and publication biases for more severe cases. The features most suggestive of underlying thiamine deficiency in this review were haemorrhagic disc swelling, normal lumbar puncture opening pressure and MRI scans showing T2/FLAIR changes in the thalamus, periaqueductal grey matter, and/or mamilliary bodies. This is consistent with known descriptions of MRI findings in thiamine deficiency [87]. Vision loss was also a common complaint. Importantly, most patients improved rapidly with vitamin supplementation (Figure 1B, Table 3 and Table 4).

The relationship between vitamin B12 deficiency and optic disc swelling is more complex because of the known association between anaemia and IIH. Biousse et al. [11] reported 23 cases with anaemia secondary to iron deficiency (17/23), B12 ± folate deficiency (2/23) or aplastic anaemia/transient erythroblastopenia (4/23); all except one of these cases had haemoglobin levels ≤8.0 g/dL. The treatment was not reported for 2/23 patients but most of the remainder (19/21) received iron and/or blood transfusions except one with vitamin B12 deficiency and another who spontaneously improved; only 3/21 were treated with acetazolamide and 20/21 had good outcomes [11]. In this review, we excluded cases with anaemia of <8.0 g/dL because of the clear association between anaemia of any cause with IIH. However, previous studies have also shown that the severity of neurological dysfunction and megaloblastic anaemia caused by vitamin B12 deficiency are not correlated; indeed, many patients with severe neurological complications from vitamin B12 deficiency have normal haemoglobin and MCVs [88]. Therefore, vitamin B12 deficiency can cause neurological dysfunction independently of any effects on haemoglobin values. Indeed, we found that there was no obvious correlation between vitamin B12 and haemoglobin values in the 11 cases of vitamin B12 deficiency included in this review. Furthermore, 36.4% (4/11) cases who did not make a full recovery on parenteral vitamin B12 replacement had normal or only mildly reduced haemoglobin values on presentation (Table S7). This suggests that anaemia was not the primary determinant of their clinical outcome. Vitamin B12 is found in meat, fish, eggs, milk, cheese, and some fortified cereals so vegetarians and vegans risk developing B12 deficiency. Vision loss, nausea/vomiting, diplopia and headache or asymptomatic presentations were reported, lumbar puncture opening pressure was often raised when checked and neuroimaging results were generally normal. These features are all consistent with the clinical presentation of IIH and improved with vitamin supplementation (Figure 1C, Table 3 and Table 4).

Typically, cases of nutritional optic neuropathy present with subacute or chronic progressive optic atrophy and central visual loss [79]. Historically, there are epidemics of optic neuropathy with this clinical presentation that have been linked to nutritional factors. For example, the Cuban epidemic of 1992–1993 affected >50,000 people, who were not overtly malnourished but heavily dependent on a carbohydrate-rich diet of cassava root; these cases improved with intravenous infusions of B vitamins or oral multivitamins together with changes to their diet. Significantly, 10–12% of these cases initially developed optic disc swelling or hyperaemia [89]. Perhaps, optic disc swelling is an early feature of nutritional optic neuropathy, before atrophy has time to develop. Alternatively, there may be physiological and environmental factors, such as age, sex, and BMI, which interact with nutritional factors to alter the clinical presentation. Whatever the explanation, the evidence from this study suggests that nutritional deficiencies can present with optic disc swelling with or without intracranial hypertension as well as optic atrophy.

Optic nerve function is dependent on multiple micronutrients, including vitamins B2 (riboflavin), B3 (niacin), B6 (pyridoxine), and B9 (folate). Although most cases of optic disc swelling included in this systematic review were attributed to the deficiency of a single micronutrient, it is likely these cases had co-incident deficiencies in other micronutrients important to optic nerve function as a result of their limited intake or malabsorption syndrome. Some tests for these other micronutrients are not readily available or easy to arrange, so deficiencies are easily missed. From a practical perspective, patients with optic disc swelling caused by diets restricted to a limited variety of foods or limited intake because of nausea, vomiting or malabsorption may benefit more from combined multivitamin supplements rather than simply supplementing the deficiencies that have been proven biochemically.

## 5. Conclusions

In summary, the findings of this study suggest that patients with IIH or unexplained optic disc swelling ought to be investigated for deficiencies in vitamin A, B1 and B12 caused by poor diet, inadequate intake or malabsorption, as early detection and treatment could prevent visual loss and other complications. Obesogenic diets of highly processed carbohydrates and meat provide lower quantities of thiamine and other micronutrients compared to fresh and/or fortified equivalents [90], which could explain the high prevalence of nutritional deficiencies among obese individuals [10].

### Future Directions

Nutritional deficiencies do not currently feature in the diagnostic or exclusion criteria for IIH. The results of this study suggest that IIH patients ought to be screened for deficiencies in vitamin A, thiamine and B12, regardless of their BMI, as early detection and treatment could prevent visual loss and other complications. Furthermore, malabsorption syndromes such as celiac disease ought to be considered as a cause of nutritional deficiencies in these patients. Although we do not know the true prevalence of micronutrient deficiencies in the IIH population, this evidence suggests that IIH management should focus on better nutrition, and not just calorie restriction, for weight loss. Further research in this area is needed.

## Figures and Tables

**Table 1:** Established associations with IIH.

**Exogenous factors**	**Antibiotics**: tetracyclines and derivatives, quinolones, e.g., nalidixic acid **Vitamin A and derivatives**: isotretinoin, all-transretinoic acid **Hormonal agents**: corticosteroid withdrawal, oral combined contraceptive pill, levonorgestrel implant, levothyroxine, tamoxifen, growth hormone, danazol, testosterone **Other**: ciclosporin, lithium, indomethacin, cimetidine
**Endogenous factors**	**Haematological**: anaemia, polycythaemia, thrombocythaemia **Venous outflow obstruction**: cerebral venous sinus thrombosis, superior vena cava obstruction, increased right-sided heart pressure **Renal**: chronic kidney disease/renal failure **Endocrine**: obesity, Addison’s disease/adrenal insufficiency, Cushing’s syndrome, hypoparathyroidism, hypothyroidism, hyperthyroidism, polycystic ovary syndrome **Respiratory**: obstructive sleep apnoea syndrome, chronic obstructive pulmonary disease, psittacosis **Autoimmune**: systemic lupus erythematosus **Nutritional**: hypervitaminosis A, iron deficiency

**Table 2:** Patient characteristics and causes of nutritional deficiencies.

	Vitamin A (n = 12)	Vitamin B1 (n = 40)	Vitamin B12 (n = 11)	Presumed (n = 2)	Whole series (n = 65)
**Patient characteristics**					
(i) Sex					
Male (M) (n, %)	9M (75%)	4M (10.0%)	9M (81.8%)	0M (0%)	22M (33.9%)
Female (F) (n, %)	3F (25%)	36F (90.0%)	2F (18.2%)	2F (100%)	43F (66.1%)
Pregnant females (n, %)	0/3F (0%)	15/36F (41.7%)	0/2F (0%)	0/2F (0%)	15/43 (34.8%)
(ii) Mean age (range)	10.2 (3–27 years)	28.1 (11–56 years)	23.0 (15 months–47 years)	14.0 (14 years)	23.4 (15 months–56 years)
**Cause of deficiency**					
(i) Malabsorption (n, %)	4/12 (33.3%)	15/40 (37.5%)	0/11 (0%)	2/2 (100%)	21/65 (32.3%)
Post-surgical (n, %)	1/12 (8.3%)	11/15 (73.3%)	0 (0%)	0/2 (0%)	12/65 (18.5%)
Gastro-intestinal pathology (n, %)	3/12 (25.0%)	4/15 (26.7%)	0 (0%)	2/2 (100%)	9/65 (13.8%)
(ii) Dietary (%)	8/12 (66.6%)	25/40 (62.5%)	9/11 (81.8%)	0/2 (0%)	42/65 (64.6%)
Hyperemesis gravidarum (n, %)	0/8 (0%)	15/25 (60.0%)	0/10 (0%)	0 (0%)	15/65 (23.1%)
Nausea and vomiting (n, %)	0/8 (0%)	5/25 (20.0%)	0/10 (0%)	0 (0%)	5/65 (7.7%)
Reduced intake (n, %)	8/8 (100%)	5/25 (20.0%)	9/9 (100%)	0 (0%)	22/65 (33.8%)
(iii) Undetermined (%)	0/12 (0%)	0/12 (0.0%)	2/11 (18.2%)	0 (0%)	2/65 (3.0%)

**Table 3:** Clinical and investigation results compared against IIH diagnostic criteria.

IIH Diagnostic Criteria	Vitamin A (n = 12)	Vitamin B1 (n = 40)	Vitamin B12 (n = 11)	Presumed (n = 2)	Whole Series (n = 65)
(i) Papilledema (n, %)	12/12 (100%)	40/40 (100%)	11/11 (100%)	2/2 (100%)	65/65 (100%)
(ii) Normal neurological examination * (n, %)	11/12 (91.7%)	3/40 (7.5%)	11/11 (100%)	2/2 (100%)	30/65 (46.2%)
(iii) Reported Lumbar puncture results (n, %)	10/12 (83.3%)	11/40 (27.5%)	04/11 (36.4%)	2/2 (100%)	25/65 (38.5%)
Raised opening pressure (n, %)	7/10 (70.0%)	0/11 (0%)	3/4 (75.0%)	2/2 (100%)	12/27 (44.4%)
Normal opening pressure (n, %)	3/10 (30.0%)	11/11 (100%)	1/4 (25.0%)	0/0 (0%)	15/27 (55.6%)
Normal CSF constituents (n, %)	10/10 (100%)	11/11 (100%)	4/4 (100%)	2/2(100%)	25/25 (100%)
(iv) Reported neuro-imaging results (n, %)	11/12 (91.7%)	32/40 (80.0%)	10/11 (90.9%)	2/2 (100%)	55/65 (84.8%)
No abnormality (n, %)	9/11 (81.8%)	7/32 (21.9%)	8/10 (80.0%)	2/2 (100%)	26/55 (47.3%)
Other pathological features (n, %)	2/11 (18.2%)	25/32 (78.1%)	2/10 (20.0%)	0/2 (0%)	29/55 (52.7.2%)
Cases meeting IIH diagnostic criteria of definite or probable IIH (n, %)	11/12 (91.7%)	3/40 (7.5%)	11/11 (100%)	2/2 (100%)	27/65 (41.5%)

* Normal neurological examination except for cranial nerve abnormalities [4].

**Table 4:** Therapeutic interventions and outcomes.

	Vitamin A (n = 12)	Vitamin B1 (n = 40)	Vitamin B12 (n = 11)	Presumed (n = 2)	Whole Series (n = 65)
**Nutritional supplementation (n, %)**	**12/12 (100%)**	**40/40 (100%)**	**11/11 (100%)**	**2/2 (100%)**	**65/65 (100%)**
Oral (n, %)	12/12 (100%)	0/40 (0%)	0/11 (0%)	2/2 (100%)	14/65 (21.5%)
Parenteral (n, %)	0/12 (0%)	39/40 (97.5%)	11/11 (100%)	0/2 (0%)	50/65 (76.9%)
Not reported (n, %)	0/12 (0%)	1/40 (2.5%)	0/11 (0%)	0/2 (0%)	1/65 (1.5%)
**Other interventions**					
Acetazolamide (n, %)	2/12 (16.7%)	0/40(0%)	1/11 (9.1%)	2/2 (100%)	4/65(6.2%)
**Clinical outcomes**					
Full recovery (n, %)	6/12 (50.0%)	31/40 (77.5%)	7/11 (63.6%)	2/2 (100%)	46/65 (70.8%)
Residual visual defects (n, %)	6/12 (50.0%)	4/40 (10.0%)	4/11 (36.4%)	0/2 (0%)	14/65 (21.5%)
Residual neurological defects (n, %)	0/12 (0%)	5/40 (12.5%)	0/11 (0%)	0/2 (0%)	5/65 (7.7%)
Death (n, %)	0/12 (0%)	4/40 (10.0%)	0/11 (0%)	0/2 (0%)	5/65 (7.7%)
Maternal death (n, %)	-	2/15 pregnancies (13.3%)	-	-	-
Fetal death (n, %)	-	5/15 pregnancies (33.3%)	-	-	-

## Data Availability

All data from this study are available in the manuscript and Supplementary Materials.

## Supplementary Materials

The following supporting information can be downloaded at: https://www.mdpi.com/article/10.3390/nu14153068/s1, Figure S1: Preferred Reporting Items for Systematic Reviews and Meta-Analyses (PRISMA) flow diagram of the search strategy and screening of results; Figure S2: Risk of bias assessment against CARE guidelines; Table S1: Search strategy for MEDLINE: National Library of medicine; Table S2: Reference list of papers excluded at full text review stage; Table S3: Cases presenting with optic disc swelling associated with vitamin A deficiency from inadequate intake; Table S4: Cases presenting with optic disc swelling associated with vitamin A deficiency from malabsorption; Table S5: Cases presenting with optic disc swelling associated with inadequate intake of vitamin B1; Table S6: Cases presenting with optic disc swelling associated with vitamin B1 deficiency from malabsorption; Table S7: Cases presenting with optic disc swelling associated with vitamin B12 deficiency; Table S8: Cases presenting with optic disc swelling associated with unknown/presumed vitamin deficiencies; Table S9: List of journals and their principal specialty audience.
