# Supplementary material for: Micronutrient Deficiencies Presenting with Optic Disc Swelling Associated with or without Intracranial Hypertension: A Systematic Review
Source: Nutrients. 2022 Jul 26;14(15):3068. doi: 10.3390/nu14153068 (PMC9331791; doi:10.3390/nu14153068)
Supplement: Supplementary file 1 [file nutrients-14-03068-s001.zip › nutrients-1827353-supplementary.pdf]

Supplementary online only materials

Contents (page number)

2:	Figure S1: Preferred Reporting Items for Systematic Reviews and Meta-Analyses (PRISMA) flow diagram of the search strategy and screening of results
3:	Figure S2: Risk of bias assessment against CARE guidelines
4:	Table S1. Search strategy for MEDLINE: National Library of medicine
5-6:	Table S2. Reference list of papers excluded at full text review stage
7:	Table S3. Cases presenting with optic disc swelling associated with vitamin A deficiency from inadequate intake
8:	Table S4. Cases presenting with optic disc swelling associated with vitamin A deficiency from malabsorption
9-13:	Table S5. Cases presenting with optic disc swelling associated with inadequate intake of vitamin B1
14-15:	Table S6. Cases presenting with optic disc swelling associated with vitamin B1 deficiency from malabsorption
16:	Table S7. Cases presenting with optic disc swelling associated with vitamin B12 deficiency
17-18:	Table S8. Cases presenting with optic disc swelling associated with unknown/presumed vitamin deficiencies
19-20:	Table S9: List of journals and their principal specialty audience
21-29:	References

Figure S1: Preferred Reporting Items for Systematic Reviews and Meta-Analyses (PRISMA) flow diagram of the search strategy and screening of results

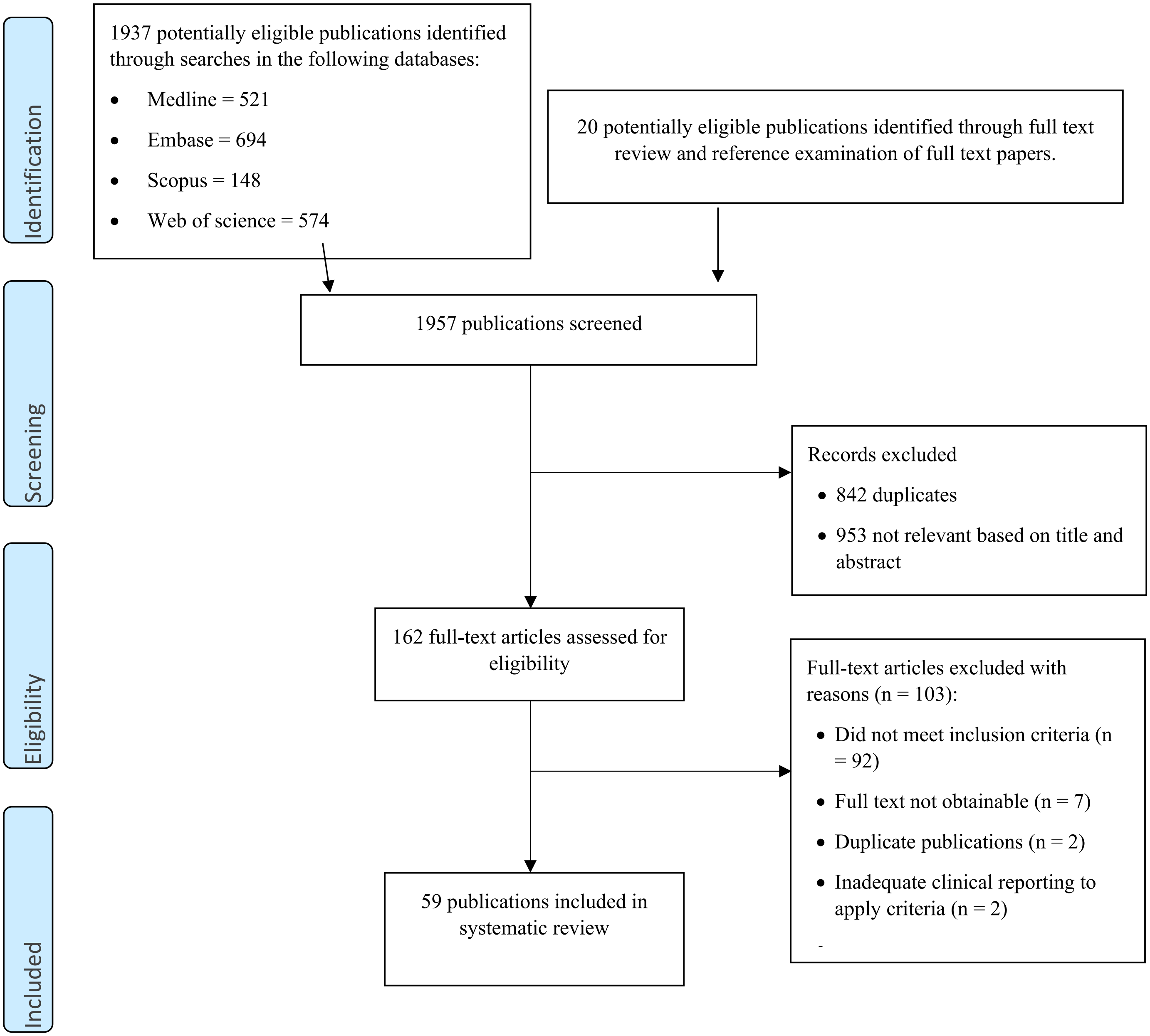

Figure S2: Risk of bias assessment against CARE guidelines

Fifty-nine manuscripts met the eligibility criteria for systematic review and were assessed for their risk of bias against CARE guidelines. Percentages reflect the proportion of manuscripts with clear documentation against each CARE criterion.

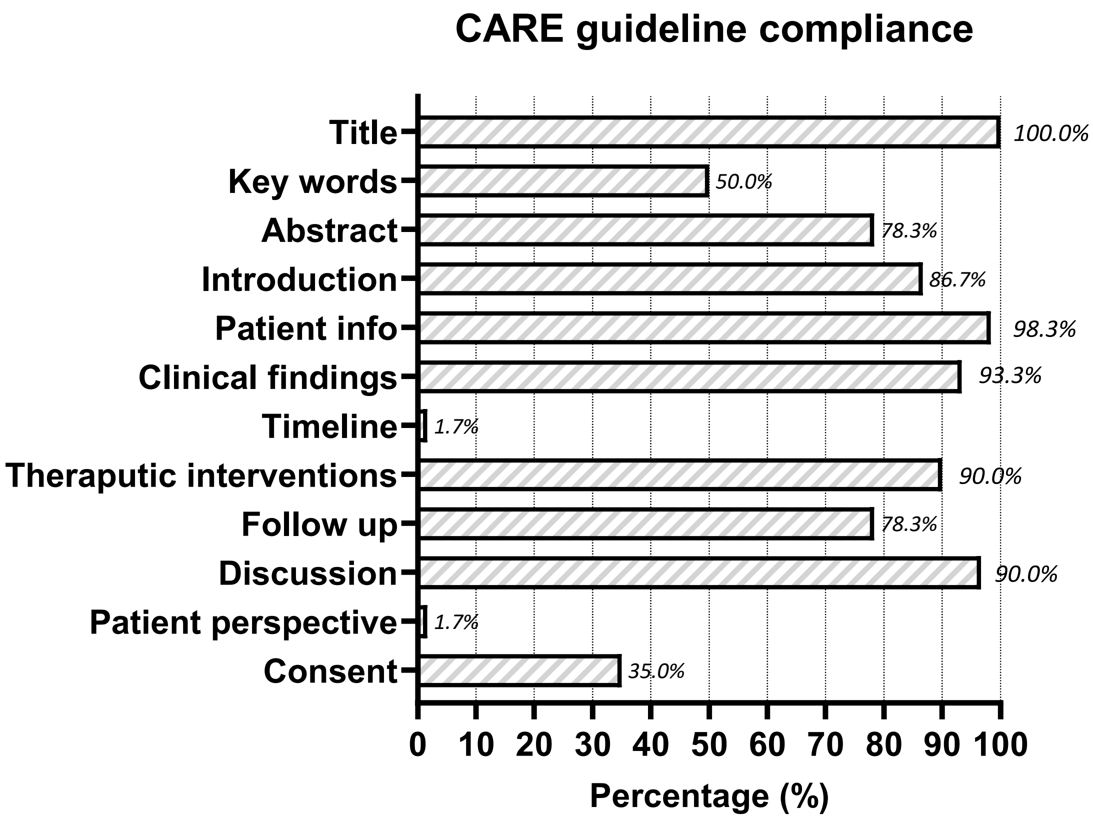

Table S1. Search strategy for MEDLINE: National Library of medicine

Search history	Search terms	Search fields
#1	Nutritional	ab,kw,ot,ti
#2	Malnutrition	ab,kw,ot,ti
#3	Diet	ab,kw,ot,ti
#4	Folate	ab,kw,ot,ti
#5	b12	ab,kw,ot,ti
#6	Cobalamin	ab,kw,ot,ti.
#7	pernicious anaemia	ab,kw,ot,ti
#8	Thiamine	ab,kw,ot,ti
#9	Vitamin	ab,kw,ot,ti
#10	Niacin	ab,kw,ot,ti
#11	Copper	ab,kw,ot,ti
#12	Pyridoxine	ab,kw,ot,ti
#13	vitamins	ab,kw,ot,ti
#14	Bariatric	ab,kw,ot,ti
#15	optic neuropathy	ab,kw,ot,ti
#16	idiopathic intracranial hypertension	ab,kw,ot,ti
#17	IIH	ab,kw,ot,ti
#18	pseudotumor cerebri	ab,kw,ot,ti
#19	Papilledema	ab,kw,ot,ti
#20	Papilloedema	ab,kw,ot,ti
#21	disc swelling	ab,kw,ot,ti
#22	disc oedema	af,kw,ot,ti
#23	disc edema	af,kw,ot,ti
#24	disk oedema	af,kw,ot,ti
#25	disk edema	af,kw,ot,ti
#26	1 or 2 or 3 or 4 or 5 or 6 or 7 or 8 or 9 or 10 or 11 or 12 or 13 or 14	
#27	15 or 16 or 17 or 18 or 19 or 20 or 21 or 22 or 23 or 24 or 25	
#28	26 and 27	

521 papers were identified with this strategy when searches were conducted on the 18th of December 2020.
Abbreviations: Abstract (ab), Key words (kw), Original title (ot), Title (ti), and All fields (af).

Table S2. Reference list of papers excluded at full text review stage.

"Unable to obtain full text" defined as inability to obtain full text manuscript despite extensive searches of online databases, local library, and google scholar. "Inadequate clinical findings" refer to publications with insufficient detail to apply inclusion/exclusion criteria. Two cases were duplicated in other publications.[158,159]

[1]	Did not meet inclusion/exclusion criteria	[2]	Did not meet inclusion/exclusion criteria
[3]	Did not meet inclusion/exclusion criteria	[4]	Did not meet inclusion/exclusion criteria
[5]	Did not meet inclusion/exclusion criteria	[6]	Did not meet inclusion/exclusion criteria
[7]	Did not meet inclusion/exclusion criteria	[8]	Did not meet inclusion/exclusion criteria
[9]	Did not meet inclusion/exclusion criteria	[10]	Did not meet inclusion/exclusion criteria
[11]	Did not meet inclusion/exclusion criteria	[12]	Did not meet inclusion/exclusion criteria
[13]	Did not meet inclusion/exclusion criteria	[14]	Did not meet inclusion/exclusion criteria
[15]	Did not meet inclusion/exclusion criteria	[16]	Did not meet inclusion/exclusion criteria
[17]	Did not meet inclusion/exclusion criteria	[18]	Did not meet inclusion/exclusion criteria
[19]	Did not meet inclusion/exclusion criteria	[20]	Did not meet inclusion/exclusion criteria
[21]	Did not meet inclusion/exclusion criteria	[22]	Did not meet inclusion/exclusion criteria
[23]	Did not meet inclusion/exclusion criteria	[24]	Did not meet inclusion/exclusion criteria
[25]	Did not meet inclusion/exclusion criteria	[26]	Did not meet inclusion/exclusion criteria
[27]	Did not meet inclusion/exclusion criteria	[28]	Did not meet inclusion/exclusion criteria
[29]	Did not meet inclusion/exclusion criteria	[30]	Did not meet inclusion/exclusion criteria
[31]	Did not meet inclusion/exclusion criteria	[32]	Did not meet inclusion/exclusion criteria
[33]	Did not meet inclusion/exclusion criteria	[34]	Did not meet inclusion/exclusion criteria
[35]	Did not meet inclusion/exclusion criteria	[36]	Did not meet inclusion/exclusion criteria
[37]	Did not meet inclusion/exclusion criteria	[38]	Did not meet inclusion/exclusion criteria
[39]	Did not meet inclusion/exclusion criteria	[40]	Did not meet inclusion/exclusion criteria
[41]	Did not meet inclusion/exclusion criteria	[42]	Did not meet inclusion/exclusion criteria
[43]	Did not meet inclusion/exclusion criteria	[44]	Did not meet inclusion/exclusion criteria
[45]	Did not meet inclusion/exclusion criteria	[46]	Did not meet inclusion/exclusion criteria
[47]	Did not meet inclusion/exclusion criteria	[48]	Did not meet inclusion/exclusion criteria
[49]	Did not meet inclusion/exclusion criteria	[50]	Did not meet inclusion/exclusion criteria
[51]	Did not meet inclusion/exclusion criteria	[52]	Did not meet inclusion/exclusion criteria

53	Did not meet inclusion/exclusion criteria	54	Did not meet inclusion/exclusion criteria
55	Did not meet inclusion/exclusion criteria	56	Did not meet inclusion/exclusion criteria
57	Did not meet inclusion/exclusion criteria	58	Did not meet inclusion/exclusion criteria
59	Did not meet inclusion/exclusion criteria	60	Did not meet inclusion/exclusion criteria
61	Did not meet inclusion/exclusion criteria	62	Did not meet inclusion/exclusion criteria
63	Did not meet inclusion/exclusion criteria	64	Did not meet inclusion/exclusion criteria
65	Did not meet inclusion/exclusion criteria	66	Did not meet inclusion/exclusion criteria
67	Did not meet inclusion/exclusion criteria	68	Did not meet inclusion/exclusion criteria
69	Did not meet inclusion/exclusion criteria	70	Did not meet inclusion/exclusion criteria
71	Did not meet inclusion/exclusion criteria	72	Did not meet inclusion/exclusion criteria
73	Did not meet inclusion/exclusion criteria	74	Did not meet inclusion/exclusion criteria
75	Did not meet inclusion/exclusion criteria	76	Did not meet inclusion/exclusion criteria
77	Did not meet inclusion/exclusion criteria	78	Did not meet inclusion/exclusion criteria
79	Did not meet inclusion/exclusion criteria	80	Did not meet inclusion/exclusion criteria
81	Did not meet inclusion/exclusion criteria	82	Did not meet inclusion/exclusion criteria
83	Did not meet inclusion/exclusion criteria	84	Did not meet inclusion/exclusion criteria
85	Did not meet inclusion/exclusion criteria	86	Did not meet inclusion/exclusion criteria
87	Did not meet inclusion/exclusion criteria	88	Did not meet inclusion/exclusion criteria
89	Did not meet inclusion/exclusion criteria	90	Did not meet inclusion/exclusion criteria
91	Did not meet inclusion/exclusion criteria	92	Did not meet inclusion/exclusion criteria
93	Unable to obtain full text		
94	Unable to obtain full text		
95	Unable to obtain full text		
96	Unable to obtain full text		
97	Unable to obtain full text		
98	Unable to obtain full text		
99	Unable to obtain full text		
100	Case duplicate: Ref 9 (main text)		
101	Case duplicate: Ref 35 (main text)		
102	Inadequate clinical findings		
103	Inadequate clinical findings		

Table S3. Cases presenting with optic disc swelling associated with vitamin A deficiency from inadequate intake

Sex	Age (years)	Weight (kg) / BMI	Clinical presentation	Duration of symptoms	Visual acuity	Optic discs	Visual field results	MRI/CT findings	Lumbar puncture (cm CSF)	Primary micronutrient deficiency (normal range)	Cause	Treatment	Response to treatment	REF
M	11	Not reported	Vision loss	Several months	6/15 RE <6/60 LE	BE swollen and pale	RE mild VF loss, LE severely constricted VF	Thickened optic nerve sheaths	Normal constituents (30)	Vitamin A= 6 (30-120 µg/dL)	Dietary	Oral vitamin A + oral acetazolamide 250mg	6/15 RE, <6/60 LE Optic disc swelling progressed to optic atrophy	104
M	12	Not reported	Vision loss	4 months	<6/60 RE HM LE	BE swollen and pale	BE constricted VF	Normal	Normal constituents (40)	Vitamin A= 5 (15-55 µg/dL)	Dietary	Oral vitamin A	<6/60 RE, HM LE Optic disc swelling progressed to optic atrophy	104
M	7	Not reported	Not reported	Not reported	6/7·5 BE	BE swollen	Not reported	Normal	Normal constituents (25)	Vitamin A= 23 (30-120 µg/dL)	Undetermined	Oral vitamin A	6/7·5 BE	104
F	10	Not reported	Not reported	Not reported	6/6 BE	BE swollen	Not reported	Normal	Normal constituents (43)	Vitamin A= 16 (30-120 µg/dL)	Undetermined	Oral vitamin A	6/6 BE	104
M	12	61.3KG BMI not reported	Vision loss, Left RAPD, rod-cone dysfunction on electrophysiology	Two weeks	6/12 RE HM LE	BE swollen	BE centrocaecal scotoma	Not reported	Minor CSF protein elevation, otherwise normal (45)	Vitamin A< 0·5 (0·92-1·71 µmol/L)	Dietary	Oral vitamin A	6/6 RE, CF LE, repeat visual electrophysiology showed resolution of changes at 3 months	105
M	14	Not reported	Vision loss, erythematous rash, dry conjunctiva with Bitot's spots, rod dysfunction on electroretinogram	5 days	6/7·5 BE	BE swollen	Not reported	Normal	Normal constituents and pressure (not reported)	Vitamin A <0·1 µmol/L (normal range not reported)	Dietary (restrictive eating)	Oral vitamin A	Vision and electrophysiology improved within one month	106
M	10	Not reported	Vision loss, corneal and conjunctival keratinization, Bitot's spots	2 months	HM BE	BE swollen	Not reported	Bony hypertrophy of the optic canals	Not reported	Vitamin A< 0·1 (0.9-1.7 µmol/L)	Dietary (restrictive eating)	Oral vitamin A	6/12 RE, 6/9 LE. Vision and corneal changes improved within one week	107
M	6	Not reported	Vision loss, corneal ulceration, facial nerve palsies, retinal dysfunction on electroretinogram	3 months	Not reported	BE swollen	Not reported	Normal	Normal constituents and pressure (not reported)	Vitamin A= 0·16 (0.9-1.7 µmol/L)	Dietary (restrictive eating)	Oral multivitamin supplementation	Visual acuity remained low, slow resolution of ocular surface and facial palsy	108

Abbreviations: M=male; F=female; RE=right eye; LE=left eye; BE=both eyes; VA=visual acuity; VF=visual field; CF=count fingers; HM=hand movement

Table S4. Cases presenting with optic disc swelling associated with vitamin A deficiency from malabsorption

Sex	Age (years)	Weight (kg) / BMI	Clinical presentation	Duration of symptoms	Visual acuity	Optic discs	Visual field results	MRI/CT findings	Lumbar puncture (cm CSF)	Primary micronutrient deficiency (normal range)	Cause	Treatment	Response to treatment	REF
M	3	Not reported	Headache	Not reported	6/6 BE	BE swollen	Not reported	Normal	Normal constituents (40)	Vitamin A= 20 (30-120 µg/dL)	Coeliac disease	Oral vitamin A + oral acetazolamide 125mg	6/6 BE	104
M	4	Not reported	Not reported	Not reported	6/6 BE	BE swollen	Not reported	Normal	Normal constituents (37)	Vitamin A= 26 (30-120 µg/dL)	Coeliac disease	Oral vitamin A	6/6 BE	104
F	27	Not reported	Vision loss<br>Ocular pain/conjunctival hyperemia	2 months	6/7·5 RE<br>6/15 LE	BE swollen + flecked retina	RE enlarged blind spot, LE diffuse VF loss	Normal	Not reported	Vitamin A= 0·8 (1·3 to 2·6 µmol/l	Biliopancreatic bypass	Oral vitamin A	6/6 BE. Visual acuity improved within 6 weeks	109
F	6	Not reported	Vision loss<br>Headache	6 months	Not reported	BE swollen	Not reported	Normal	Normal constituents (50)	Vitamin A= 45·3 (350-960 µgram/ml)	Cystic fibrosis	Oral vitamins, pancreatic enzyme supplementation	Vision remained low in the left eye	110

Abbreviations: M=male; F=female; RE=right eye; LE=left eye; BE=both eyes; VA=visual acuity; VF=visual field; HM=hand movements

Table S5. Cases presenting with optic disc swelling associated with inadequate intake of vitamin B1

Sex	Age (years)	Weight (kg) / BMI	Clinical presentation	Duration of symptoms	Visual acuity	Optic discs	Visual fields	MRI/CT findings	Lumbar puncture (cm CSF)	Primary micronutrient deficiency (normal range)	Cause/ gestational age	Fetal / Maternal outcome	Treatment	Response to treatment	REF
F	42	Not reported	Vision loss Confusion Ataxia	3 days	Not reported	BE swollen + peripapillary haems	Not reported	Normal	Not reporter	Not reported	Hyperemesis gravidarum/ 16 weeks	Fetal death	IV thiamine	Death 4 days after admission	111
F	26	Not reported	Vision loss Ophthalmoplegia Nystagmus Ataxia	2 days	6/36 RE 6/18 LE	RE swollen, LE peripapillary haems	Not reported	Thalamic/ periaqueductal changes on MRI FLAIR	Not reporter	Not reported	Hyperemesis gravidarum/ 20 weeks	Fetal death	IV thiamine	VA and optic disc improved after 3 days	112
F	17	Not reported	Vision loss Confusion Ophthalmoplegia Nystagmus Ataxia	9 days	Not reported	BE swollen + peripapillary haems	Not reported	Bilateral Thalamic MRI hyperintensities on MRI FLAIR	Normal constituents (13)	Not reported	Hyperemesis gravidarum/ 14 weeks	Fetal death	IV thiamine	Improved over days	113
F	22	Not reported	Vision loss Confusion Ophthalmoplegia Ataxia	several days	6/36 RE 6/18 LE	BE swollen + peripapillary haems	Not reported	Thalamic changes on T2 /DWI weighted MRI	Normal constituents ("normal")	Thiamine=64 (66-200 nmol/L)	Hyperemesis gravidarum/ 16 weeks	Healthy baby delivered at term	IV thiamine	VA improved in 3 days and other symptoms recovered	114
F	29	Not reported	Vision loss Confusion Ophthalmoplegia Ataxia	15 days	CF BE	BE swollen + peripapillary haems	Not reported	Bilateral Thalamic/ mamillary body changes on MRI FLAIR	Not reporter	Not reported	Hyperemesis gravidarum/ 18 weeks	Healthy baby delivered at term	IV thiamine	VA improved in 3 days and normal in 2 weeks	115
F	24	Not reported	Vision loss Confusion Ophthalmoplegia Nystagmus Ataxia	Not reported	Not reported	BE swollen + peripapillary haems	Not reported	Normal CT scan/venogram	Normal constituents (19)	Thiamine pyrophosphate 355% (2-20%)	Hyperemesis gravidarum/ 16 weeks	Healthy baby delivered at term	IV thiamine	6/5 BE. Rapid improvement within hours	116

Sex	Age (years)	Weight (kg) / BMI	Clinical presentation	Duration of symptoms	Visual acuity	Optic discs	Visual fields	MRI/CT findings	Lumbar puncture (cm CSF)	Primary micronutrient deficiency (normal range)	Cause/ gestational age	Fetal / Maternal outcome	Treatment	Response to treatment	REF
F	29	Not reported	Confusion Ophthalmoplegia Nystagmus Ataxia	Not reported	Not reported	BE swollen	Not reported	Thalamic/ caudate/ periaqueductal changes on MRI	Not reporter	Not reported	Hyperemesis gravidarum/ not reported	Healthy baby delivered at term	IV thiamine	Fundal findings resolved by 3 months, residual ataxia/nystagmus	117
F	25	Not reported	Vision loss Confusion Ophthalmoplegia Nystagmus Ataxia	several weeks	Not reported	BE swollen	Not reported	Bilateral Thalamic/ periaqueductal grey changes on MRI FLAIR and T2	Not reporter	Not reported	Hyperemesis gravidarum/ 22 weeks	Healthy baby delivered by Caesarean section at 30 weeks	IV thiamine	Patient died after caesarean section due to central pontine myelinolysis	118
F	29	Not reported	Vision loss Confusion Ophthalmoplegia Nystagmus Ataxia	11 days	Not reported	BE swollen	Not reported	Bilateral Thalamic/ periaqueducts hyperintensities on MRI T2	Not reporter	Not reported	Hyperemesis gravidarum/ 20 weeks	Healthy baby delivered by Caesarean section at 31 weeks	IV thiamine	Slow recovery over months	119
F	27	Not reported	Vision loss Confusion Nystagmus Ataxia	11 days	Not reported	BE swollen + peripapillary haems	Not reported	Bilateral Thalamic hyperintensities on T2 MRI	Not reporter	Not reported	Hyperemesis gravidarum/ 12 weeks	Healthy baby delivered by Caesarean section at 34 weeks	IV thiamine	Rapid improvement in ocular signs and full recovery in 2 weeks	120
F	21	71Kg	Vision loss Confusion Ophthalmoplegia Nystagmus Ataxia	10 days	HM	BE swollen + peripapillary haems	Not able to conduct	Normal MRI	Not reporter	Not reported	Hyperemesis gravidarum/ 17 weeks	Fetal death	IV thiamine	Rapid improvement within one day	121

Sex	Age (years)	Weight (kg) / BMI	Clinical presentation	Duration of symptoms	Visual acuity	Optic discs	Visual fields	MRI/CT findings	Lumbar puncture (cm CSF)	Primary micronutrient deficiency (normal range)	Cause/ gestational age	Fetal / Maternal outcome	Treatment	Response to treatment	REF
F	32	Not reported	Vision loss<br>Nystagmus<br>Ataxia	2 weeks	6/18 RE<br>6/12 LE	BE swollen + peripapillary haems	Not reported	Thalamic/ periaqueductal hyperintensity on MRI FLAIR and T2	Not conducted	Not reported	Hyperemesis gravidarum/ 15 weeks	Not reported	IV thiamine	Significant clinical improvement within one day	122
F	34	Not reported	Vision loss<br>Nystagmus	3 weeks	LP RE<br>CF LE	BE swollen	Not reported	Thalamic/ periaqueductal changes on MRI FLAIR	Not reporter	Not reported	Hyperemesis gravidarum/ Not reported	Not reported	IV thiamine	VA improved within 3 days	123
F	31	Not reported	Vision loss<br>Nystagmus<br>Ataxia	2 days	RE LP<br>LE 6/18	BE swollen<br>Peripapillary haems	Not reported	Not reported	Not reporter	Not reported	Hyperemesis gravidarum/ 16 weeks	Fetal death	IV thiamine	Rapid improvement within 5 days, complete within 6 weeks	124
F	25	Not reported	Vision loss<br>Ophthalmoplegia<br>Nystagmus<br>Ataxia	3 days	BE 6/60	BE swollen<br>Peripapillary haems	Not reported	Hyperintensities in dorso-medial thalamus on DWI	Not reporter	Not reported	Hyperemesis gravidarum/ 20 weeks	Fetus survived event, no birth outcome reported.	IV thiamine	Rapid improvement within 24 hours, complete within one week	125
F	34	Not reported	Vision loss<br>Confusion<br>Ataxia	12 weeks	Not reported	BE swollen	Not reported	Normal	Norma Constituents (“normal”)	Not reported	Reduced intake due to depression	Not pregnant	Thiamine (no route specified)	VA and other symptoms resolved in 4 months	126
F	56	BMI 15	Vision loss<br>Nystagmus	1 week	CF BE	Thickening of RNFL	RE reduced sensitivity<br>LE central scotoma	Normal	Not reporter	Thiamine= 38 (70-200 nmol/L)	Reduced intake after dental extraction	Not pregnant	IV thiamine	6/5 BE Dramatic visual improvement within 2 days	127

Sex	Age (years)	Weight (kg) / BMI	Clinical presentation	Duration of symptoms	Visual acuity	Optic discs	Visual fields	MRI/CT findings	Lumbar puncture (cm CSF)	Primary micronutrient deficiency (normal range)	Cause/ gestational age	Fetal / Maternal outcome	Treatment	Response to treatment	REF
F	20	Not reported	Confusion Nystagmus	Not reported	Not reported	Thickening of RNFL	Not reported	Not reported	Not reporter	Not reported	Reduced intake due to alcoholism	Not pregnant	IV thiamine	Persistent cognitive impairment and vision loss, fundal findings resolved	127
F	28	Not reported	Vision loss Ophthalmoplegia Nystagmus Ataxia	10 days	Not reported	BE swollen	Not reported	Thalamic changes on MRI	Not reporter	Thiamine= 15 (24-66 nmol/l)	Reduced intake due to alcoholism	Not pregnant	IV thiamine, then oral thiamine	VA improved with 3 weeks	128
F	33	Not reported	Confusion Ophthalmoplegia Ataxia	Not reported	Not reported	BE swollen	Not reported	Not reported	Not reporter	Not reported	Vomiting due to gastroparesis	Not pregnant	IV thiamine	Symptoms improved in 10 days	129
F	39	BMI 29	Vision loss Confusion Ophthalmoplegia Nystagmus Ataxia	7 days	<6/60 BE	BE swollen + peripapillary haems	BE dense centrocaecal scotoma	Thalamic/ mamillary body changes on T2 MRI	Normal Constituents (15)	Thiamine= 8 (8-30 nmol/L)	Nausea & vomiting	Not pregnant	IV thiamine	6/7·5 RE, 6/15 LE. Rapid improvement in vision in 2 days. Persistent ataxia	130
F	23	Not reported	Headache Vision loss Ophthalmoplegia	Not reported	Not reported	BE swollen	Not reported	T2 hyperintensities	Normal constituents (19)	Thiamine= 3 (8-30 nmol/L)	Nausea & vomiting	Not pregnant	IV thiamine	Symptoms resolved in 2 weeks	131
F	46	Not reported	Visual loss Confusion Ophthalmoplegia Ataxia	9 days	LP BE	BE swollen + peripapillary haems	Not reported	Thalamic/caudate changes on MRI	Normal constituents (7)	Thiamine= 25 (80-150 nmol/L)	Nausea caused by chemotherapy for oral cancer	Not pregnant	IV thiamine, then oral thiamine	6/7·5 RE, 6/9 LE. VA improved within 4 days	132

Sex	Age (years)	Weight (kg) / BMI	Clinical presentation	Duration of symptoms	Visual acuity	Optic discs	Visual fields	MRI/CT findings	Lumbar puncture (cm CSF)	Primary micronutrient deficiency (normal range)	Cause/ gestational age	Fetal / Maternal outcome	Treatment	Response to treatment	REF
F	11	Not reported	Vision loss<br>Confusion<br>Nystagmus<br>Ataxia	2 days	6/60 RE<br>6/24 LE	BE swollen + peripapillary haems	Not reported	Thalamic/mamillary body changes on MRI FLAIR	Not reporter	Not reported	Restrictive diet	Not pregnant	IV thiamine	VA improved rapidly, ataxia and colour vision resolved in 2 weeks	133
M	22	Not reported	Vision loss<br>Nystagmus<br>Ophthalmoplegia	2 days	<6/60 BE	BE swollen	Not reported	Thalamic and mamillary body hyperintensity on T2	Not reporter	Thiamine= 11 (20-50 ng/dL)	Anorexia post-surgery	Not pregnant	IV thiamine	Complete recovery within 4 weeks	134

Abbreviations: M=male; F=female; RE=right eye; LE=left eye; BE=both eyes; VA=visual acuity; VF=visual field; HM=hand movements; RNFL=retinal nerve fibre layer

f

Table S6. Cases presenting with optic disc swelling associated with vitamin B1 deficiency from malabsorption

Sex	Age (years)	Weight (kg) / BMI	Clinical presentation	Duration of symptoms	Visual acuity	Optic discs	Visual fields	MRI/CT findings	Lumbar puncture (cm CSF)	Primary micronutrient deficiency (normal range)	Cause	Treatment	Response to treatment	REF
M	29	Not reported	Visual loss<br>Confusion<br>Ataxia	7 days	NPL	BE swollen + peripapillary haems	Not reported	Not reported	Normal constituent ("had not increased")	Not conducted	Chronic diarrhoea & principally carbohydrate diet	IV thiamine	VA improved to CF in 12 hours then 2/60, oedema improved within 2 weeks	135
M	12	Not reported	Confusion<br>Ophthalmoplegia<br>Nystagmus<br>Ataxia	Not reported	Not reported	BE swollen	Not reported	Thalamic/ periaqueductal grey matter changes on T2	Not reported	Not reported	Amoebic dysentery	IV thiamine	Symptoms resolved within 4 days	136
M	35	Not reported	Visual loss<br>Ophthalmoplegia	Not reported	6/36 RE<br>6/30 LE	BE swollen + peripapillary haems	BE dense central scotoma	Not reported	Not reported	Not reported	Ulcerative colitis & poor intake	IV thiamine	6/6 BE. Rapid improvement in vision in 2 days, VF changes and optic atrophy remained	9
F	24	Not reported	Vision loss<br>Nystagmus<br>Ataxia	10 days	3/60 RE<br>6/60 LE	BE swollen + peripapillary haems	Incongruent VF loss	Thalamic changes on MRI FLAIR	Normal constituents (19)	Not reported	Gastric bypass	IV thiamine	6/6 BE. Rapid improvement within 24 hours.	137
F	11	Not reported	Confusion	Several days	Not reported	BE swollen + peripapillary haems	Not reported	Thalamic/ putamen/ frontal lobe changes on MRI FLAIR	Normal constituent and pressure (not reported)	Not reported	Ileal bypass surgery	IV thiamine	VA and ocular changes resolved after 5 days	138
F	3	Not reported	Vomiting<br>Nystagmus	4 days	Not reported	BE swollen + peripapillary haems	Not reported	Hyperintensity of the caudate and putamen on MRI T2	Not reported	Not reported	Ileal resection due to ascariasis	IV thiamine	Death occurred within 2 months	139
F	17	Not reported	Vision loss<br>Confusion<br>Ataxia	3 weeks	Not able	BE swollen + peripapillary haems	Not reported	Thalamic/ periaqueductal changes on MRI FLAIR	Not reported	"Undetectable"	Pancreatitis & low intake	IV thiamine	Resolved rapidly	140
F	24	114kg	Confusion<br>Nystagmus<br>Ophthalmoplegia<br>Ataxia	Not reported	Not reported	BE swollen + peripapillary haems	Not reported	Not reported	Not reported	Not reported	Roux-en-y bypass	IV thiamine	Recovery of ocular findings. Cognitive changes persisted. Patient committed suicide	141

Sex	Age (years)	Weight (kg) / BMI	Clinical presentation	Duration of symptoms	Visual acuity	Optic discs	Visual fields	MRI/CT findings	Lumbar puncture (cm CSF)	Primary micronutrient deficiency (normal range)	Cause	Treatment	Response to treatment	REF
F	35	Not reported	Vision loss<br>Ophthalmoplegia<br>Nystagmus<br>Ataxia	5 days	6/18 RE<br>6/15 LE	BE swollen	BE dense central scotoma	Normal	Not reported	Not reported	Roux-en-y bypass	IV thiamine	VA 6/6. Rapid improvement within hours	142
F	37	Not reported	Vision loss<br>Ataxia	Unknown duration	3/60 RE<br>6/60 LE	BE swollen + peripapillary haems	Not reported	Not reported	Not reported	Thiamine=13<br>(38-122 g/L)	Roux-en-y bypass	IV thiamine	6/7·5 RE, 6/6 LE. VA improved rapidly to normal after one week	143
F	47	Not reported	Vision loss<br>Confusion<br>Ophthalmoplegia<br>Nystagmus<br>Ataxia	3 days	Not reported	BE swollen	Not reported	Normal	Not reported	Not reported	Roux-en-y bypass & poor pre-operative intake	Thiamine supplementati on of parenteral nutrition	VA 6/24 RE, 6/15 LE. Slow improvement over 2 weeks	144
F	22	Not reported	Vision loss<br>Confusion<br>Ophthalmoplegia<br>Nystagmus<br>Ataxia	Not reported	6/12 RE,<br>6/7·5 LE	BE swollen, Peripapillary haems	Not reported	Thalamic/ mamillary body hyperintensity on T2/FLAIR	Not reported	Thiamine=6.0<br>(4.5-15.1 nmol/L)	Roux-en-y bypass	IV thiamine	Full resolution of symptoms in 4 weeks	145
F	29	Not reported	Vision loss<br>Nystagmus<br>Ophthalmoplegia	1 week	Not reported	BE swollen + peripapillary haems	Not reported	Prominent optic nerve sheaths	Normal constituent and pressure (21)	Not reported	Sleeve gastrectomy	IV thiamine	Symptoms improved rapidly	146
F	35	Not reported	Vision loss<br>Confusion<br>Ataxia	8 weeks	Not reported	BE swollen	Not reported	Thalamic/ periaqueductal changes on MRI FLAIR	Not reported	Thiamine=2<br>(4-15 nmol/L)	Sleeve gastrectomy	IV thiamine	Patient did not recover and died	147
F	37	BMI 35	Vision loss<br>Confusion<br>Nystagmus<br>Ataxia	10 weeks	6/6 BE	BE swollen + peripapillary haems	Not reported	Not reported	Not reported	Thiamine=13.9<br>(32-95 µgram/L)	Sleeve gastrectomy	IV thiamine	Fundal findings resolved by 3 months	148

Abbreviations: M=male; F=female; RE=right eye; LE=left eye; BE=both eyes; VA=visual acuity; VF=visual field; CF=Count fingers; HM=hand movements; RNFL=retinal nerve fibre layer; BMI=Body mass index; FLAIR=Fluid attenuated inversion recovery; IV=Intravenous

Table S7. Cases presenting with optic disc swelling associated with vitamin B12 deficiency.

Sex	Age (years)	Weight (kg) / BMI	Clinical presentation	Duration	Visual acuity	Optic discs	Visual fields	MRI /CT findings	Lumbar puncture (cm CSF)	Primary micronutrient deficiency (normal range)	Associated micronutrient deficiencies and test results (normal range)	Cause	Treatment	Response to treatment	REF
F	11	Not reported	Visual loss	2 weeks	LP RE<br>6/60 LE	BE swollen	Not reported	Normal	Not reported	Vitamin B12= "severe deficiency"	Vitamin A= "deficient"<br>Macrocytic anaemia (values not reported)	Dietary deficiency	IM vitamin B12<br>Oral multivitamins	VA remained poor and discs become atrophic	149
F	14	68kg / 27.6	Visual loss	Not reported	Not reported	Be swollen	Not reported	Normal	Normal constituents (>30)	Vitamin B12=123 (180-900 pg/mL) =32% reduction<br>Homocysteine= 19 (5-15 μmol/L)	Hb=12·5g/dl (normal range not reported)<br>MCV=82 fL (normal range not reported)<br>Hypersegmented neutrophils	Vegetarianism	IM vitamin B12	Slow improvement	150
M	47	Not reported	Visual loss	2 weeks	6/120 BE	BE swollen + peripapillary haems	BE reduced sensitivity, centrocaecal scotoma of one eye	Normal	Not reported	Vitamin B12= 100 (200-900 ng/ml) =50% reduction	Hb= 10 g/dl (13·5 to 17·5) =26% reduction<br>MCV=139·6 (80 to 100 fL)<br>Thiamine=1·5 (2·5-7·5 ng/ml)<br>Negative for Intrinsic Factor	Inadequate intake from alcoholism	IM vitamin B12<br>Oral multivitamins	Vision recovered to 6/6 BE	151
M	13	Not reported	Headache<br>Nausea<br>Diplopia<br>Vision loss	1 month	Not reported	BE swollen	Not reported	Normal	Normal constituents (43)	Vitamin B12= 137 (197-866 pg/ml) =30% reduction<br>Homocysteine=9.2 (4·3-9·9 pg/ml)	Hb=8·4 g/dl (normal range not reported)<br>MCV=94·4 (normal range not reported)<br>Hypersegmented neutrophils on smear	Dietary deficiency	IM vitamin B12 & Acetazolamide	Improvement after one week	152
M	18	62kg / 21	Vison loss	12 weeks	6/240 BE	BE swollen	Centrocaecal scotoma	Normal	Not reported	Vitamin B12= 165 (254-1320 pg/ml) =35% reduction	Hb=13·9 (14-18 g/dL) =1% reduction<br>Folic acid=1·7 (3·1-17·5 ng/ml)	Restrictive diet	IM vitamin B12<br>Oral Folate	VA improved in one eye	153
M	15 Months	Not reported	Vomiting<br>Esotropia	One week	Not reported	BE swollen	Not reported	Diffuse cerebral atrophy	Normal constituents (31)	Vitamin B12= 210 (251-910 pg/ml) =16% reduction<br>Homocysteine= 22.79 (3.3-11.3 μmol/L)	Hb=8.8 g/dL (normal range not reported)<br>Macrocytic anaemia (MCV not reported)	Vegetarianism in mother	IM vitamin B12	Full recovery	154
M	28	Not reported	Visual loss	Not reported	6/60 BE	Be swollen	Enlarged blind spots	Normal	Not reported	Vitamin B12= 155 (200-835 pg/ml) =23% reduction	Hb=9.2 g/dl (normal range not reported)<br>MCV=122.8 (78-100 fL)	Vegetarianism	IM vitamin B12	VA improved to 6/6 BE	155

Sex	Age (years)	Weight (kg) / BMI	Clinical presentation	Duration	Visual acuity	Optic discs	Visual fields	MRI /CT findings	Lumbar puncture (cm CSF)	Primary micronutrient deficiency (normal range)	Associated micronutrient deficiencies and test results (normal range)	Cause	Treatment	Response to treatment	REF
M	19	Not reported	Asymptomatic	Not reported	6/12 RE<br>6/9 LE	BE swollen	Not reported	Normal	Not reported	Vitamin B12= 52 (180-1000 ng/l) =71% reduction	Hb=9.9g/dl (normal range not reported)<br>Folic acid=1.9 (>3 µgram/l)<br>MCV=111.1 fl (normal range not reported)	Restrictive diet	IM vitamin B12	VA improved to 6/6 BE and disc swelling resolved	156
M	29	Not reported	Vision loss, headache	7 weeks	3/60 RE<br>6/24 LE	BE swollen + hyperemic	Not reported	Not reported	Not reported	Vitamin B12= 80 (145-596 pmol/L) =45% reduction	Other bloods "unremarkable"	Vegetarianism	IM vitamin B12	Mild improvement in vision to 6/12 RE, 6/24 LE	157
M	34	Not reported	Vision loss	4 weeks	CF BE	RE swollen, LE atrophic	RE inferior VF loss<br>LE dense VF loss	Normal	Normal constituents (18)	Vitamin B12= 200 (243-894 pg/ml) =18% reduction	Hb=14.4 (13.5-18 g/dL) =7% above lower limit<br>MCV=81.3 (81-98 fL)	Undetermined	IM vitamin B12	VF improved but remained CF BE	158
M	39	Not reported	Visual loss	Sudden	6/30 RE<br>6/6 LE	RE swollen	RE inferior altitudinal defect	Ischemic lesions on MRI	Not reported	Vitamin B12= 187 pg/ml (normal range not reported)<br>Homocysteine= 15 µmol/L (normal range not reported)	Complete blood count and other "biochemical investigations", were normal	Undetermined	IM vitamin B12	VA and field changes improved in one week	159

Elevated levels of Homocysteine (HC) and methylmalonic acid (MMA) are biochemical indicators of functional vitamin B12 deficiency. Hypersegmented neutrophils can be an early sign of vitamin B12 or folate deficiency

Abbreviations: M=male; F=female; RE=right eye; LE=left eye; BE=both eyes; VA=Snellen visual acuity; VF=visual field; LP=light perception; CF=counting fingers; HM=hand movements; IM=intramuscula

Table S8. Cases presenting with optic disc swelling associated with unknown/presumed vitamin deficiencies

Sex	Age (years)	Weight (Kg) / BMI	Presenting symptoms	Duration of symptoms	Visual acuity	Optic discs	Visual fields	MRI /CT findings	Lumbar puncture (cm CSF)	Primary micronutrient deficiency (normal range)	Cause	Treatment	Response to treatment	REF
F	14	72kg / 30	Vision loss Headache	1 year	6/60 RE 6/12 LE	BE swollen	Not reported	Normal	Normal constituents (29)	?	Coeliac disease	Gluten free diet Acetazolamide	6/6 BE. Findings resolved within 2 months	[160]
F	14	41.8kg / 18.1	Headache	6 months	Not reported	BE swollen	Not reported	Normal	Normal constituents (36)	?	Coeliac disease	Gluten free diet Acetazolamide	Rapid improvement in symptoms	[161]

Abbreviations: M=male; F=female; RE=right eye; LE=left eye; BE=both eyes; VA=visual acuity; VF=visual field; HM=hand movements

Table S9: List of journals and their principal specialty audience

References presented as per main text bibliography. Each journal defined as principally directed at Neurology (Neuro), Ophthalmology (Ophth), and Other journals. Where not clearly defined (as per the journal of Neuro-ophthalmology) choice based upon greater proportion of self-defined specialty origin in the target audience (Ophthalmology , in this example[162]).

Reference number	Journal name	Journal specialty	Visual acuity included	Classification
1	Journal of child neurology	Neurology/Paediatric	Yes	Neuro
2	Annal of clinical case reports	General medicine/all	Yes	Other
3	Journal of paediatric ophthalmology/strabismus	Ophthalmology/Paediatric	Yes	Ophth
4	Paediatric child health	Paediatric	Yes	Other
5	Development medicine child neurology	Neurology/paediatric	No	Neuro
6	American Journal of Ophthalmology	Ophthalmology/Paediatric	Yes	Ophth
7	Paediatric Neurology	Neurology/Paediatric	No	Neuro
8	Malaysian journal of medical science	General medicine	No	Other
9	Internet Journal of Genomics/Proteomics	General medicine	Yes	Other
10	Nature clinical practice of Neurology	Neurology	Yes	Neuro
11	Medical journal of Australia	General medicine	Yes	Other
12	Oman Journal of Ophthalmology	Ophthalmology	Yes	Neuro
13	Postgrad medical journal	General medicine	No	Other
14	Journal of postgraduate medicine	General medicine	No	Other
15	Indian journal of critical medical care	General medicine	No	Other
16	Journal of Maternal-Fetal Neonatal medicine	Obstetric/Neonatal	No	Other
17	Obstetric and Gynaecological Survey	Obstetric/Gynaecological	No	Other
18	The Lancet	General medicine	Yes	Other
19	Cureus	General medicine	Yes	Other
20	Journal of family medicine and primary care	General medicine	Yes	Other
21	British medical Journal	General medicine	Yes	Other
22	Journal of the association of physicians India	General medicine	Yes	Other
23	Journal of General medicine internal Medicine	General medicine	No	Other
24	Journal of Neuro-ophthalmology	Ophthalmology/Neurology	Yes	Ophth
25	Journal of General medicine internal Medicine	General medicine	Yes	Other
26	Neurology	Neurology	No	Neuro
27	Clinical ophthalmology (Auckland/ NZ)	Ophthalmology	Yes	Ophth
28	Neurology	Neurology	No	Neuro
29	Neurology	Neurology	Yes	Neuro
30	Eye	Ophthalmology	Yes	Ophth
31	Internal medicine	General medicine	Yes	Other
32	Journal of the Formosan medical association	General medicine	Yes	Other
33	Paediatric Radiology	Paediatric/Radiology	No	Other
34	Documenta Ophthalmologica	Ophthalmology	Yes	Ophth
35	Survey Ophthalmology	Ophthalmology	Yes	Ophth
36	Paediatric emergency care	Paediatric	No	Other
37	Paediatric neurology	Neurology/paediatric	No	Neuro

Reference number	Journal name	Journal specialty	Visual acuity included	Classification
38	Neurology	Neurology	Yes	Neuro
39	Gastroenterology	Gastroenterology	No	Other
40	Seminars in Ophthalmology	Ophthalmology	Yes	Ophth
41	Oschner	General medicine	Yes	Other
42	Medical principles and practice (Kuwait)	General medicine	No	Ophth
43	Journal of Neuro-ophthalmology	Ophthalmology/Neurology	Yes	Ophth
44	Neurology	Neurology	No	Neuro
45	Neurology	Neurology	No	Neuro
46	Case reports in Ophthalmology	Ophthalmology	Yes	Ophth
47	International Ophthalmology	Ophthalmology	Yes	Ophth
48	Paediatric haematology/oncology	Paediatric/oncology	No	Other
49	Optometry and visual science	Ophthalmology	Yes	Ophth
50	European journal of General medicine	General medicine	No	Other
51	Eating and weight disorders	Nutrition	Yes	Other
52	Indian journal of paediatrics	Paediatrics	No	Other
53	Taiwan journal of Ophthalmology	Ophthalmology	Yes	Ophth
54	BMJ case reports	Ophthalmology	Yes	Ophth
55	Annal of medicine and surgery	General medicine	Yes	Other
56	Iranian red crescent medical journal	General medicine	Yes	Other
57	BMJ case reports	General medicine	Yes	Other
58	Gastroenterology	Gastroenterology	Yes	Other
59	Journal of paediatric child health	Paediatric	No	Other

References

(1) Hmaied, W.; Ben Khalifa, M. M.; Miled, W.; Kamoun, H.; Ksontini, I.; Sakka, S.; El Fekih, L. [Ocular Findings in Megaloblastic Anemia Associated with Diabetes. A Case Report]. *Complicat. Ocul. Assoc. Anemie Biermer Diabete Propos Un Cas* **2005**, *83* (5), 305–307.

(2) Ghonim, H.; Elwan, S.; El Dokla, A. Acute Motor and Sensory Axonal Neuropathy Following Diarrheal Illness with Concomitant Dry Beri Beri Following Bariatric Surgery. *Neurology* **2019**, *92* (15 Supplement 1).

(3) Alves, I.; Silva, M. J.; Fraga, C. Optic Neuropathy in Pernicious Anaemia. *Eur. J. Neurol.* **2016**, *23*, 450–450.

(4) Glueck, C. J.; Aregawi, D.; Goldenberg, N.; Golnik, K. C.; Sieve, L.; Wang, P. Changes in Papilledema, Headache,and Life Status in Response to Diet and Metformin in Women with Idiopathic Intracranial Hypertension with and without Concurrent Polycystic Ovary Syndrome or Hyperinsulinemia. *J. Investig. Med.* **2005**, *53* (2), S396–S397.

(5) Sadun, A. A.; Martone, J. F.; Muci-Mendoza, R.; Reyes, L.; DuBois, L.; Silva, J. C.; Roman, G.; Caballero, B. Epidemic Optic Neuropathy in Cuba. Eye Findings. *Arch. Ophthalmol. Chic. Ill 1960* **1994**, *112* (5), 691–699.

(6) Golnik, K. C.; Schaible, E. R. Folate-Responsive Optic Neuropathy. *Journal of Neuro-Ophthalmology*, 1994, *14*, 163–169.

(7) Tucker, K.; Hedges, T. R. Food Shortages and an Epidemic of Optic and Peripheral Neuropathy in Cuba. *Nutr. Rev.* **1993**, *51* (12), 349–357.

(8) Gorgulu, U.; Ergun, U.; Kavak, P.; Inan, L. E.; Yoldas, T. K. “Subacute Combined Degeneration” Due to Vitamin B12 Deficiency: A Case Review. *Gazi Medical Journal*, 2016, *27*, 32–36.

(9) van Noort, B. A.; Bos, P. J.; Klopping, C.; Wilmink, J. M. Optic Neuropathy from Thiamine Deficiency in a Patient with Ulcerative Colitis. *Doc. Ophthalmol. Adv. Ophthalmol.* **1987**, *67* (1–2), 45–51.

(10) Greber-Platzer, S.; Walleczek, N.; Breu, M.; Seidl, R. Case Report: 13 Year Old Obese Girl Presenting with Neurological Symptoms. *Obesity Facts*, 2018, *11*, 215.

(11) Zwart, S. R.; Gibson, C. R.; Gregory, J. F.; Mader, T. H.; Stover, P. J.; Zeisel, S. H.; Smith, S. M. Astronaut Ophthalmic Syndrome. *FASEB J. Off. Publ. Fed. Am. Soc. Exp. Biol.* **2017**, *31* (9), 3746–3756.

(12) Haydek, J. P.; To, M. Q.; Lora, M. H. Eying Other Causes. *J. Gen. Intern. Med.* **2017**, *32* (2 Supplement 1), S496–S497.

(13) Sirdofsky, M.; Kattah, J.; Macedo, P. Intracranial Hypertension in a Dieting Patient. *J. Neuroophthalmol.* **1994**, *14* (1), 9–11.

(14) Hochman, H. I.; Mejlszenkier, J. D. Cataracts and Pseudotumor Cerebri in an Infant with Vitamin D-Deficiency Rickets. *J. Pediatr.* **1977**, *90* (2), 252–254.

(15) Schmitt, R. A.; Denton, W. J. Tobacco-Alcohol Amblyopia: Combined Toxic and Nutritional Optic Neuropathy. *Clinical and Refractive Optometry*, 2010, *21*, 208–212.

(16) Hoyt, C. S.; Billson, F. A. Low-Carbohydrate Diet Optic Neuropathy. *Med. J. Aust.* **1977**, *1* (3), 65–66.

(17) Rosling, H. Unbalanced Diet behind the Epidemic on Cuba. Smoking as a Risk Factor of Optic Neuropathy. *Läkartidningen*, 1994, *91*, 4018–4019.

(18) Hsu, C. T.; Miller, N. R.; Wray, M. L. Optic Neuropathy from Folic Acid Deficiency without Alcohol Abuse. *Ophthalmol. J. Int. Ophtalmol. Int. J. Ophthalmol. Z. Augenheilkd.* **2002**, *216* (1), 65–67.

(19) Roman, G. C. Epidemic Neuropathy in Cuba: A Plea to End the United States Economic Embargo on a Humanitarian Basis. *Neurology* **1994**, *44* (10), 1784–1786.

(20) Humbertjean-Selton, L.; Selton, J.; Riou-Comte, N.; Lacour, J. C.; Mione, G.; Richard, S. Bilateral Optic Neuropathy Related to Severe Anemia in a Patient with Alcoholic Cirrhosis: A Case Report and Review of the Literature. *Clinical and Molecular Hepatology*, 2018, *24*, 417–423.

(21) McCartney, F.; Berkenstock, M.; Ackert, J. M. Vitamin A Deficiency as a Cause of Optic Neuropathy. *Invest. Ophthalmol. Vis. Sci.* **2014**, *55* (13).

(22) Inaba, M.; Torii, T.; Shinoda, K.; Yamasaki, R.; Ohyagi, Y.; Kira, J. [Peripheral Neuropathy, Myelopathy, Cerebellar Ataxia, and Subclinical Optic Neuropathy Associated with Copper Deficiency Occurring 23 Years after Total Gastrectomy]. *Rinsho Shinkeigaku* **2011**, *51* (6), 412–416.
(23) Eells, J. T.; Gonzalez-Quevedo, A.; McMartin, K. E.; Sadun, A. A. Folate-Deficiency and Elevated Serum and CSF Formate Concentrations in Patients with Cuban Epidemic Optic Neuropathy. *Investigative Ophthalmology and Visual Science*, 1996, *37*.
(24) Isen, D. R.; Kline, L. B. Neuro-Ophthalmic Manifestations of Wernicke Encephalopathy. *Eye and Brain*, 2020, *12*, 49–60.
(25) Martinez, J.; de Letona, L. Optic Neuropathy in Vitamin B12 Deficiency. *Lancet 352* (9122), 147–147.
(26) Jaiser, S. R.; Winston, G. P. Subacute Combined Degeneration of the Spinal Cord despite Prophylactic Vitamin B12 Treatment. *Journal of Clinical Neuroscience*, 2012, *19*, 1607–1607.
(27) Adachi, T.; Suzuki, K.; Kumagai, N.; Nishida, T. A Case of Optic Neuropathy Due to Malnutrition and Presumed Thiamine Deficiency. *Japanese Journal of Clinical Ophthalmology*, 2004, *58*, 607–611.
(28) Kaisari, E.; Roemer, S.; Minghelli, G.; Kawasaki, A. B12 Deficiency, Optic Neuropathy and Cyanocobalamin Nasal Spray. *Acta Ophthalmologica*, 2020, *98*, e923–e924.
(29) Ahn, T. B.; Cho, J. W.; Jeon, B. S. Unusual Neurological Presentations of Vitamin B(12) Deficiency. *Eur. J. Neurol.* **2004**, *11* (5), 339–341.
(30) Kim, S.; Park, J.; Lee, S.; Chun, J. Nutritional Optic Neuropathy Due to Folic Acid Deficiency. *Journal of Korean Ophthalmological Society*, 2020, *61*, 1235–1239.
(31) Al Nababteh, A.; Young, A. C.; Sheeran, P. Severe Chronic Vitamin A Toxicity in an Infant Receiving Dietary Supplements. *Clinical Toxicology*, 2012, *50*, 657–658.
(32) Klevay, L. M. Ocular Lesions from Copper Deficiency. *Indian Journal of Medical Research*, 2017, *146*, 430-+.
(33) Alemayehu, W. Pseudotumor Cerebri (Toxic Effect of the “Magic Bullet”). *Ethiop. Med. J.* **1995**, *33* (4), 265–270.
(34) Larner, A. J. Missed Diagnosis of Vitamin B-12 Deficiency Presenting with Paraesthetic Symptoms. *Int. J. Clin. Pract.* **2002**, *56* (5), 377–378.
(35) Aleyasin, A.; Ghazanfari, M.; Houshmand, M. Leber Hereditary Optic Neuropathy: Do Folate Pathway Gene Alterations Influence the Expression of Mitochondrial DNA Mutation? *Iranian Journal of Public Health*, 2010, *39*, 53–60.
(36) Larner, A. J. Visual Failure Caused by Vitamin B12 Deficiency Optic Neuropathy. *International Journal of Clinical Practice*, 2004, *58*, 977–978.
(37) Ali, M. S.; Mutahar, E.; Al Hashmi, H.; Al Sagheir, A. Pseudotumor Cerebri Associated with All-Trans Retinoic Acid Treatment in Female Patients with Acute Promyelocytic Leukemia: A Case Series. *Biol. Blood Marrow Transplant.* **2015**, *21* (2 SUPPL. 1), S185.
(38) Lopez-Hern; ez, N.; Garcia-Escriva, A.; Pampliega-Perez, A.; Alvarez-Sauco, M.; Martin-Estefania, C.; Asensio-Asensio, M. [Peripheral and Optical Myeloneuropathy in a Folic Acid Deficient Alcoholic Patient]. *Mieloneuropatia Periferica Opt. En Un Paciente Alcohol. Deficiente En Acido Folico* **2003**, *37* (8), 726–729.
(39) Amrani, Y.; Terriza, F.; Moreno, C.; Casado, A. Pseudotumor Cerebri Caused by Vitamin Excess Due to Intake of Milk Enriched with A and D Vitamins. Letter. *Revista de neurologia*, 1996, *24*, 1304–1305.
(40) Moskowitz, Y.; Leibowitz, E.; Ronen, M.; Aviel, E. Pseudotumor Cerebri Induced by Vitamin A Combined with Minocycline. *Annals of ophthalmology*, 1993, *25*, 306–308.
(41) Asad, Z.; Chaudhary, A.; Awab, A. Reversible Blindness Associated with Diabetic Ketoacidosis: A Rare Combination. *Journal of Investigative Medicine*, 2016, *64*, 975–976.
(42) Mroczkowski, M. M.; Redgrave, G. W.; Miller, N. R.; McCoy, A. N.; Guarda, A. S. Reversible Vision Loss Secondary to Malnutrition in a Woman with Severe Anorexia Nervosa, Purging Type, and Alcohol Abuse. *Int. J. Eat. Disord.* **2011**, *44* (3), 281–283. https://doi.org/10.1002/eat.20806.
(43) Aytekin, E.; Caglar, N. S.; Ozgonenel, L.; Tutun, S.; Kurkcuoglu, A. Vitamin B12 Deficiency-Related Myelopathy: Case Report. *Turkiye Klinikleri Tip Bilimleri Dergisi*, 2012, *32*, 290–293.
(44) Murphy, T. E.; Costanzi, J. J. Pseudotumor Cerebri Associated with Pernicious Anemia. *Annals of internal medicine*, 1969, *70*, 777–782.

(45) Badrane, N.; Abbada, A.; Chaoui, H.; Aoued, L.; Rhalem, N.; Benjelloune, B. S.; Bencheikh, R. S. Blindness Following Closantel Poisoning: Report of Three Cases. *Clinical Toxicology*, 2013, *51*, 280.
(46) Naismith, R. T.; Shepherd, J. B.; Weihl, C. C.; Tutlam, N. T.; Cross, A. H. Acute and Bilateral Blindness Due to Optic Neuropathy Associated with Copper Deficiency. *Archives of Neurology*, 2009, *66*, 1025–1027.
(47) Bannink, N.; Joosten, K. F. M.; van Veelen, M. L. C.; Bartels, M. C.; Tasker, R. C.; van Adrichem, L. N. A.; Van der Meulen, J.; Vaandrager, J. M.; de Jong, T. H. R.; Mathijssen, I. M. J. Papilledema in Patients with Apert, Crouzon, and Pfeiffer Syndrome: Prevalence, Efficacy of Treatment, and Risk Factors. *J. Craniofac. Surg.* **2008**, *19* (1), 121–127.
(48) Oe, K.; Kishi, T. Nutritional Optic Neuropathy in a Case of Pernicious Anemia and Subacute Combined Degeneration of the Spinal Cord. *Japanese Journal of Clinical Ophthalmology*, 2010, *64*, 517–520.
(49) Bechetoille, A.; Allain, P.; Ebran, J. M.; Mauras, Y. [Modifications in Blood Zinc and Lead Concentrations and ALA-Dehydratase Activity in Optic Neuropathy from Alcohol and Tobacco Abuse]. *Modif. Conc. Sang. Zinc Plomb Act. ALA-Dehydratase Au Cours Neuropathies Opt. Alcoolo-Tabagiques* **1983**, *6* (3), 231–235.
(50) O'Mahony, J.; Shroff, M.; Banwell, B. Mimics and Rare Presentations of Pediatric Demyelination. *Neuroimaging Clin. N. Am.* **2013**, *23* (2), 321–336.
(51) Bektas, O.; Yilmaz, A.; Teber, S.; Aksoy, E.; Deda, G. Pseudotumor Cerebri Associated with Cod Liver Oil Use: Case Report. *Turk. Klin. Pediatri* **2013**, *22* (3), 143–145.
(52) Palmero-Fern; ez, L.; Fern; ez-Treguerres, F.; Santos-Bueso, E.; Saenz-Frances, F.; Martinez-de-la-Casa, J. M.; Garcia-Feijoo, J.; Garcia-Sanchez, J. Hyperhomocysteinemia in Chronic Alcoholism: A Case with Retinal Manifestations. *Arch. Soc. Espanola Oftalmol.* **2014**, *89* (8), 336–339.
(53) Benrabah, R.; Lumbroso, L.; Limon, S.; Hamard, H.; Morin, Y. Unexplained bilateral optic disk oedema: So called idiopathic, drug induced, intracranial hypertension must be evoked. *J. Fr. Ophtalmol.* **1995**, *18* (4), 282–285.
(54) Pantalon, A. D.; Danielescu, C.; Chiselita, D. Combined Etiology for Bilateral and Simultaneous Optic Neuropathy in a Patient with Ciancobalamin Deficit and Hepatitis C Treated with Peg-Interferon and Ribavirin. *Romanian J. Ophthalmol.* **2016**, *60* (3), 188–194.
(55) Bhaskar, H.; Chaudhary, R. Vitamin B12 Deficiency Due to Chlorofluorocarbon: A Case Report. *Case reports in medicine*, 2010, *2010*, 691563.
(56) Pineles, S. L.; Avery, R. A.; Liu, G. T. Vitamin B-12 Optic Neuropathy in Autism. *Pediatrics*, 2010, *126*, E967–E970.
(57) Bhettay, E. M.; Bakst, C. M. Hypervitaminosis A Causing Benign Intracranial Hypertension. A Case Report. *South Afr. Med. J. Suid-Afr. Tydskr. Vir Geneeskd.* **1988**, *74* (11), 584–585.
(58) Pott, J. W. R.; van Everdingen, J. A. M.; van Schooneveld, M. J.; Vanhaelemeesch, N. Can a Vitamin B12 Deficiency Trigger the Expression of Leber Hereditary Optic Neuropathy (LHON) in Carriers? *Acta Ophthalmol. (Copenh.)* **2018**, *96*, 16–16.
(59) Bjorkenheim, B. Optic Neuropathy Caused by Vitamin-B12 Deficiency in Carriers of the Fish Tapeworm, Diphyllobothrium Latum. *Lancet Lond. Engl.* **1966**, *1* (7439), 688–690.
(60) Ramos-Levi, A. M.; Sanchez-Pernaute, A.; Herrera, M. A. R. Dermatitis and Optic Neuropathy Due to Zinc Deficiency after Malabsortive Bariatric Surgery. *Nutricion Hospitalaria*, 2013, *28*, 1345–1347.
(61) Brueckner, F.; Kohl, B.; Muehlhausen, C.; Pust, B. Pseudotumor Cerebri in an 8 Month Old Infant. *Neuropediatrics*, 2012, *43*.
(62) Rapoport, Y.; Lavin, P. J. M. Nutritional Optic Neuropathy Caused by Copper Deficiency after Bariatric Surgery. *Journal of Neuro-Ophthalmology*, 2016, *36*, 178–181.
(63) Btaiche, I. F.; Yeh, A. Y.; Wu, I. J.; Khalidi, N. Neurologic Dysfunction and Pancytopenia Secondary to Acquired Copper Deficiency Following Duodenal Switch: Case Report and Review of the Literature. *Nutrition in Clinical Practice*, 2011, *26*, 583–592.
(64) Reid, H. A.; Harris, W. Reversible Papilloedema in Pernicious Anaemia. *Br. Med. J.* **1951**, *1* (4696), 20. https://doi.org/10.1136/bmj.1.4696.20.
(65) Chacko, J. G.; Rodriguez, C. J.; Uwaydat, S. H. Nutritional Optic Neuropathy Status Post Bariatric Surgery. *Neuro-Ophthalmology*, 2012, *36*, 165–167.

(66) Santos, E.; Alvares, B.; Costa, A. P. Sudden Severe Vision Loss and Persistent Vomiting: From a Suspicion of Neuromyelitis Optica to a Diagnosis of Acute Thinner Intoxication. *Neurology* **2020**, *94* (15 Supplement).
(67) Chan, W.; Almasieh, M.; Catrinescu, M. M.; Levin, L. A. Cobalamin-Associated Superoxide Scavenging in Neuronal Cells Is a Potential Mechanism for Vitamin B12–Deprivation Optic Neuropathy. *American Journal of Pathology*, 2018, *188*, 160–172.
(68) Sawicka-Pierko, A.; Obuchowska, I.; Hady, R. H.; Mariak, Z.; Dadan, J. Nutritional Optic Neuropathy Following Bariatric Surgery. *Wideochirurgia I Inne Techniki Maloinwazyjne*, 2014, *9*, 662–666.
(69) Chavala, S. H.; Kosmorsky, G. S.; Lee, M. K.; Lee, M. S. Optic Neuropathy in Vitamin B12 Deficiency. *European Journal of Internal Medicine*, 2005, *16*, 447–448.
(70) Sedel, F.; Challe, G.; Mayer, J. M.; Boutron, A.; Fontaine, B.; Saudubray, J. M.; Brivet, M. Thiamine Responsive Pyruvate Dehydrogenase Deficiency in an Adult with Peripheral Neuropathy and Optic Neuropathy. *Journal of Neurology, Neurosurgery and Psychiatry*, 2008, *79*, 846–847.
(71) Chisholm, I. A. Serum Cobalamin and Folate in the Optic Neuropathy Associated with Tobacco Smoking. *Canadian Journal of Ophthalmology*, 1978, *13*, 105–109.
(72) Shimozono, M.; Townsend, J. C.; Ilsen, P. F.; Bright, D. C. Acute Vision Loss Resulting from Complications of Ethanol Abuse. *J. Am. Optom. Assoc.* **1998**, *69* (5), 293–303.
(73) Chu, J.; Xerras, D. A High Pressure Situation: A Rare Cause of Headache in a 37 Year-Old Man. *J. Gen. Intern. Med.* **2013**, *28* (SUPPL. 1), S257.
(74) Spinazzi, M.; Angelini, C.; Patrini, C. Subacute Sensory Ataxia and Optic Neuropathy with Thiamine Deficiency. *Nature Reviews Neurology*, 2010, *6*, 288–293.
(75) Cisse, F. A.; Bah, H.; Konate, F.; Camara, N.; Camara, M. I.; Conde, K.; Kassory, I. F. B.; Sanogo, A.; Diakhate, I.; Cisse, A. [Neurological Disorders Related to Vitamin B12 Deficiency in Prisons in Guinea: A 22-Case Study]. *Manif. Neurol. Assoc. Carence En Vitam. B12 En Milieu Carcer. En Guinee Etude 22 Obs.* **2013**, *106* (3), 156–159.
(76) Stambolian, D.; Behrens, M. Optic Neuropathy Associated with Vitamin B12 Deficiency. *American Journal of Ophthalmology*, 1977, *83*, 465–468.
(77) Clare, G.; Colley, S.; Kennett, R.; Elston, J. S. Reversible Optic Neuropathy Associated with Low-Dose Methotrexate Therapy. *J. Neuro-Ophthalmol. Off. J. North Am. Neuro-Ophthalmol. Soc.* **2005**, *25* (2), 109–112.
(78) Syed, S.; Lioutas, V. Tobacco-Alcohol Amblyopia: A Diagnostic Dilemma. *J. Neurol. Sci.* **2013**, *327* (1–2), 41–45.
(79) Coskun, M.; Sevencan, N. O. The Evaluation of Ophthalmic Findings in Women Patients With Iron and Vitamin B12 Deficiency Anemia. *Translational Vision Science & Technology*, 2018, *7*.
(80) Theodoropoulos, D. S. Optic Neuropathy in Vitamin B12 Deficiency. *Lancet Lond. Engl.* **1998**, *352* (9122), 146–147.
(81) Danesh-Meyer, H.; Kubis, K. C.; Wolf, M. A. Chiasmopathy? *Surv. Ophthalmol.* **2000**, *44* (4), 329–335.
(82) Tiwana, H. K.; Raymond, G.; Littleton, K.; Singhal, R.; Kumar, A. Thiamine Deficiency With Mashed Potatoes: A Novel Case of Wernicke Encephalopathy Manifesting With Retinal Hemorrhages in a Pediatric Patient. *Cureus*, 2020, *12*, e10429.
(83) de Silva, P.; Jayamanne, G.; Bolton, R. Folic Acid Deficiency Optic Neuropathy: A Case Report. *Journal of medical case reports*, 2008, *2*, 299.
(84) Traber, G.; Baumgartner, M. R.; Schwarz, U.; Pangalu, A.; Donath, M. Y.; Landau, K. Subacute Bilateral Visual Loss in Methylmalonic Acidemia. *J. Neuro-Ophthalmol. Off. J. North Am. Neuro-Ophthalmol. Soc.* **2011**, *31* (4), 344–346.
(85) Dobson, R.; Alvares, D. The Difficulties with Vitamin B12. *Pract. Neurol.* **2016**, *16* (4), 308–311.
(86) Vasconcellos, L. F. R.; Correa, R. B.; Chimelli, L.; Nascimento, F.; Fonseca, A. B.; Nagel, J.; Novis, S. A. P.; Vincent, M. [Myelopathy Due to Vitamin B12 Deficiency Presenting as Transverse Myelitis]. *Mielopatia Por Defic. Vitam. B12 Apresentando-Se Como Mielite Transversa* **2002**, *60* (1), 150–154.
(87) Edmunds, C.; Behrens, M.; Lewis, L.; Lennon, R. Pseudotumor Cerebri and Low Vitamin-A Intake. *JAMA: The Journal of the American Medical Association*, 1973, *226*, 674.

(88) Wheatley, T.; Clark, J. D.; Edwards, O. M.; Jordan, K. Retinal Haemorrhages and Papilloedema Due to Benign Intracranial Hypertension in a Pregnant Diabetic. *Diabet. Med. J. Br. Diabet. Assoc.* **1986**, *3* (5), 482–484.
(89) Forderreuther, S.; Straube, A. Are there nutrition-triggered headache disorders? *Aktuelle Ernahrungsmedizin* **1999**, *24* (2), 97–101.
(90) Zaki, S. A.; Lad, V.; Abdagire, N. Vitamin D Deficiency Rickets Presenting as Pseudotumor Cerebri. *J. Neurosci. Rural Pract.* **2013**, *4* (4), 464–466.
(91) Foulds, W. S.; Chisholm, I. A.; Stewart, J. B.; Wilson, T. M. The Optic Neuropathy of Pernicious Anemia. *Archives of Ophthalmology*, 1969, *82*, 427–432.
(92) Zayed, M. G.; Hickman, S. J.; Batty, R.; McCloskey, E. V.; Pepper, I. M. Unilateral Compressive Optic Neuropathy Due to Skull Hyperostosis Secondary to Nutritional Vitamin A Deficiency. *Clinical Cases in Mineral and Bone Metabolism*, 2015, *12*, 75–77.
(93) Akaji, K.; Katayama, M.; Sagoh, M.; Hirose, Y.; Murakami, H. Idiopathic intracranial hypotension associated with decreased blood concentration of vitamin A. *Brain Nerve* **1996**, *48* (12), 1135–1139.
(94) Akashi, T.; Takeuchi, S.; Iijima, Y.; Mizuki, N.; Watanabe, Y.; Kamesawa, H. Suspected Nutritional Optic Neuropathy in a Case of Crohn Disease. *Japanese Journal of Clinical Ophthalmology*, 2004, *58*, 1945–1949.
(95) Areekul, S.; Churdchu, K.; Roongpisuthipong, C.; Thanomsak, W. Optic Neuropathy in a Patient with Vitamin B12 Deficiency: A Case Report. *Journal of the Medical Association of Thailand*, 1992, *75*, 715–718.
(96) Grzybowski, A.; Pieniazek, M. Nutritional Optic Neuropathy in Patients Hospitalized for Alcohol Withdrawal. *Alcoholism: Clinical and Experimental Research*, 2014, *38*, 3068–3068.
(97) Lam, S.; Lam, B. L. Bilateral Retinal Hemorrhages from Megaloblastic Anemia: Case Report and Review of Literature. *Ann. Ophthalmol.* **1992**, *24* (3), 86–90.
(98) Suzuki, S.; Kumanomido, T. Optic Neuropathy from Thiamine Deficiency. *Intern. Med.* **1997**, *36* (7), 532.
(99) Winslet, M. C.; Donovon, I. A.; Aitchison, F. Wernicke's Encephalopathy in Association with Complicated Acute Pancreatitis and Morbid Obesity. *Br. J. Clin. Pract.* **1990**, *44* (12), 771–773.
(100) Namasivayam, B.; Arthanari, A.; Rajesh, N.; Siddharthan, V.; Ravi, E. Hyperemesis Gravidarum Induced Wernicke's Encephalopathy: Presenting as Rapidly Progressive Loss of Vision. *Ann. Indian Acad. Neurol.* **2010**, *13* (5 SUPPL. 1), S40.
(101) Singh, S.; Kumar, A. Wernicke Encephalopathy after Obesity Surgery: A Systematic Review. *Neurology* **2007**, *68* (11), 807–811.
(102) Mahase, E. Teenager Diagnosed with Optic Neuropathy Resulting from Poor Diet despite Normal BMI. *BMJ (Clinical research ed.)*, 2019, *366*, l5361.
(103) Rao, C. V.; Selhorst, J. B.; Thomas, F. P. Cobalamine Deficiency with Bilateral Optic Neuropathy, Multiple White Matter Lesions, and Normal B12 Levels. *Ann. Neurol.* **2004**, *56*, S38–S38.
(104) Dotan, G.; Goldstein, M.; Stolovitch, C.; Kesler, A. Pediatric Pseudotumor Cerebri Associated with Low Serum Levels of Vitamin A. *Journal of Child Neurology*, 2013, *28*, 1370–1377.
(105) Osunkunle, O. A.; Purbrick, R. M.; Downes, S. M. Visual Loss in Raised Intracranial Pressure Associated with Severe Vitamin A Deficiency. *Ann Clin Case Rep 2017 2 1261*.
(106) Lewis, C. D.; Traboulsi, E. I.; Rothner, A. D.; Jeng, B. H. Xerophthalmia and Intracranial Hypertension in an Autistic Child with Vitamin A Deficiency. *J. Pediatr. Ophthalmol. Strabismus* **2011**, *48 Online*, e1-3.
(107) Kinlin, L. M.; Vresk, L.; Friedman, J. N. Vision Loss in a Child with Autism Spectrum Disorder. *Paediatr. Child Health* **2019**.
(108) McSweeney, N.; Johnson, N.; Moore, W.; Robinson, R. Optic Neuritis and Facial Nerve Palsies in Autism. *Developmental Medicine and Child Neurology*, 2009, *51*, 33–34.
(109) Panozzo, G.; Babighian, S.; Bonora, A. Association of Xerophthalmia, Flecked Retina, and Pseudotumor Cerebri Caused by Hypovitaminosis A. *Am. J. Ophthalmol.* **1998**, *125* (5), 708–710.
(110) Lucidi, V.; Di Capua, M.; Rosati, P.; Papadatou, B.; Castro, M. Benign Intracranial Hypertension in an Older Child with Cystic Fibrosis. *Pediatr. Neurol.* **1993**, *9* (6), 494–495.

(111) Sulaiman, W.; Othman, A.; Mohamad, M.; Salleh, H. R.; Mushahar, L. Wernicke's Encephalopthy Associated with Hyperemesis Gravidarum - A Case Report. *Malays. J. Med. Sci. MJMS* **2002**, *9* (2), 43–46.
(112) Namasivayam, B.; Arthanari, A.; Senthilkumaran, S.; Chellammal, P.; Siddharthan, V.; Manikam, R. Loss of Vision: A Rare Presentation in Hyperemesis Gravidarum Induced Wernicke's Encephalopathy. *Internet J. Genomics Proteomics* **2012**, *6* (2), 3.
(113) Wilson, R. K.; Kuncl, R. W.; Corse, A. M. Wernicke's Encephalopathy: Beyond Alcoholism. *Nat. Clin. Pract. Neurol.* **2006**, *2* (1), 54–58.
(114) Ferdinands, M. D.; Seneviratne, J.; White, O. Visual Deterioration in Hyperemesis Gravidarum. *Med. J. Aust.* **2005**, *182* (11), 585–586. https://doi.org/10.5694/j.1326-5377.2005.tb06820.x.
(115) Mathew, N. R.; Menon, S. G.; Mathew, M. Ocular Manifestations in a Case of Wernicke's Encephalopathy Due to Hyperemesis Gravidarum. *Oman journal of ophthalmology*, 2018, *11*, 85–87.
(116) Mumford, C. J. Papilloedema Delaying Diagnosis of Wernicke's Encephalopathy in a Comatose Patient. *Postgrad. Med. J.* **1989**, *65* (764), 371–373.
(117) Ashraf, V. V.; Prijesh, J.; Praveenkumar, R.; Saifudheen, K. Wernicke's Encephalopathy Due to Hyperemesis Gravidarum: Clinical and Magnetic Resonance Imaging Characteristics. *J. Postgrad. Med.* **2016**, *62* (4), 260–263.
(118) Kantor, S.; Prakash, S.; Chandwani, J.; Gokhale, A.; Sarma, K.; Albahrani, M. J. Wernicke's Encephalopathy Following Hyperemesis Gravidarum. *Indian J. Crit. Care Med. Peer-Rev. Off. Publ. Indian Soc. Crit. Care Med.* **2014**, *18* (3), 164–166. https://doi.org/10.4103/0972-5229.128706.
(119) Di Gangi, S.; Gizzo, S.; Patrelli, T. S.; Saccardi, C.; D'Antona, D.; Nardelli, G. B. Wernicke's Encephalopathy Complicating Hyperemesis Gravidarum: From the Background to the Present. *J. Matern.-Fetal Neonatal Med. Off. J. Eur. Assoc. Perinat. Med. Fed. Asia Ocean. Perinat. Soc. Int. Soc. Perinat. Obstet.* **2012**, *25* (8), 1499–1504. https://doi.org/10.3109/14767058.2011.629253.
(120) Chiossi, G.; Neri, I.; Cavazzuti, M.; Basso, G.; Facchinetti, F. Hyperemesis Gravidarum Complicated by Wernicke Encephalopathy: Background, Case Report, and Review of the Literature: *Obstet. Gynecol. Surv.* **2006**, *61* (4), 255–268. https://doi.org/10.1097/01.ogx.0000206336.08794.65.
(121) Tesfaye, S.; Achari, V.; Yang, Y. C.; Harding, S.; Bowden, A.; Vora, J. P. Pregnant, Vomiting, and Going Blind. *The Lancet* **1998**, *352* (9140), 1594. https://doi.org/10.1016/S0140-6736(98)08325-1.
(122) Mun-Wei, L.; Gayathri, G.; Kwang Hwee, G.; Ruban, K.; Suresh Kumar, V.; Shatriah, I. Optic Discs Swelling Procrastinates Wernicke's Encephalopathy Associated with Hyperemesis Gravidarum: A Case Report and Review of Literature. *Cureus 10* (6). https://doi.org/10.7759/cureus.2793.
(123) Palakkuzhiyil, N.; Rehiman, S.; Manoj, P. P. B.; Hameed, S.; Uvais, N. A. Visual Loss and Optic Neuropathy Associated with Wernicke's Encephalopathy in Hyperemesis Gravidarum. *Journal of family medicine and primary care*, 2019, *8*, 1243–1245.
(124) Galloway, P. J. Wernicke's Encephalopathy and Hyperemesis Gravidarum. *Br. Med. J.* **1992**, *305* (6861), 1096–1096. https://doi.org/10.1136/bmj.305.6861.1096-a.
(125) Chitra, S.; Lath, K. V. S. Wernicke's Encephalopathy with Visual Loss in a Patient with Hyperemesis Gravidarum. *J. Assoc. Physicians India* **2012**, *60*, 53–56.
(126) Lulla, P. D.; Lu, L. The Deficient B-Vitamin, Causing a "Very-Very" Bizzare Presentation. *Journal of General Internal Medicine*, 2011, *26*, S537–S538.
(127) Sia, P. I.; Sia, D. I. T.; Crompton, J. L.; Casson, R. J. Nerve Fiber Layer Infarcts in Thiamine Deficiency. *J. Neuro-Ophthalmol. Off. J. North Am. Neuro-Ophthalmol. Soc.* **2015**, *35* (3), 274–276.
(128) Fujikawa, T.; Taniguchi, A.; Sogabe, Y. Significant Visual Impairment in an Alcoholic Patient. *Journal of General Internal Medicine*, 2019, *34*, S634.
(129) Lineback, C.; Brahmbhatt, N.; Abbott, S. A Case of Non-Alcoholic Wernicke's Encephalopathy and Dry Beri-Beri with GI Involvement Following a Period of Prolonged Gastrointestinal Illness. *Neurology* **2020**, *94* (15 Supplement).
(130) Gratton, S. M.; Lam, B. L. Visual Loss and Optic Nerve Head Swelling in Thiamine Deficiency without Prolonged Dietary Deficiency. *Clinical ophthalmology (Auckland, N.Z.)*, 2014, *8*, 1021–1024.
(131) Srikanth-Mysore, C.; Marky, B. Oculomotor Dysfunction Due to Idiopathic Intracranial Hypertension and Thiamine Deficiency. *Neurology* **2014**, *82* (10 SUPPL. 1).

(132) Lukas, R. V.; Piantino, J.; Nichols, J.; Cohen, E.; Haraf, D. J.; Rezania, K. Thiamine Deficiency Presenting with Encephalopathy and Optic Neuropathy after Chemoradiation for Tongue Cancer. *Neurology* **2010**, *74* (9), A466–A466.
(133) Cooke, C. A.; Hicks, E.; Page, A. B.; Mc Kinstry, S. An Atypical Presentation of Wernicke's Encephalopathy in an 11-Year-Old Child. *Eye* **2006**, *20* (12), 1418–1420. https://doi.org/10.1038/sj.eye.6702275.
(134) Suzuki, S.; Kumanomido, T. Optic Neuropathy from Thiamine Deficiency. *Internal Medicine*, 1997, *36*, 532.
(135) Yeh, W. Y.; Lian, L. M.; Chang, A.; Cheng, C. K. Thiamine-Deficient Optic Neuropathy Associated with Wernicke's Encephalopathy in Patients with Chronic Diarrhea. *Journal of the Formosan Medical Association*, 2013, *112*, 165–170.
(136) Sparacia, G.; Banco, A.; Lagalla, R. Reversible MRI Abnormalities in an Unusual Paediatric Presentation of Wernicke's Encephalopathy. *Pediatr. Radiol.* **1999**, *29* (8), 581–584. https://doi.org/10.1007/s002470050652.
(137) Kulkarni, S.; Lee, A. G.; Holstein, S. A.; Warner, J. E. A. You Are What You Eat. *Surv. Ophthalmol.* **2005**, *50* (4), 389–393.
(138) Kamasak, T.; Kul, S.; Tusat, M.; Ozgun, N.; Cansu, A. A Case of Wernicke Encephalopathy Developing after Ileal Bypass Surgery. *Pediatr. Emerg. Care* **2018**, *34* (12), e223–e225.
(139) Vasconcelos, M. M.; Silva, K. P.; Vidal, G.; Silva, A. F.; Domingues, R. C.; Berditchevsky, C. R. Early Diagnosis of Pediatric Wernicke's Encephalopathy. *Pediatr. Neurol.* **1999**, *20* (4), 289–294. https://doi.org/10.1016/s0887-8994(98)00153-2.
(140) Miller, D. Blind, Deaf and Confused: An Unusual Case of Wernicke's Encephalopathy in a Young Adult. *Neurology* **2016**, *86* (16 SUPPL. 1).
(141) Kramer, L. D.; Locke, G. E. Wernicke's Encephalopathy. Complication of Gastric Plication. *J. Clin. Gastroenterol.* **1987**, *9* (5), 549–552. https://doi.org/10.1097/00004836-198710000-00013.
(142) Longmuir, R.; Lee, A. G.; Rouleau, J. Visual Loss Due to Wernicke Syndrome Following Gastric Bypass. *Semin. Ophthalmol.* **2007**, *22* (1), 13–19.
(143) Lawton, A. W.; Frisard, N. E. Visual Loss, Retinal Hemorrhages, and Optic Disc Edema Resulting from Thiamine Deficiency Following Bariatric Surgery Complicated by Prolonged Vomiting. *Ochsner Journal*, 2017, *17*, 112–114.
(144) Gokce, M.; Bulbuloglu, E.; Tuncel, D.; Ozdemir, G.; Kale, I. T. Nonalcoholic Wernicke's Encephalopathy with Prominent Astasia and Optic Neuropathy. *Med. Princ. Pract. Int. J. Kuwait Univ. Health Sci. Cent.* **2005**, *14* (6), 438–440.
(145) Bohnsack, B. L.; Patel, S. S. Peripapillary Nerve Fiber Layer Thickening, Telangiectasia, and Retinal Hemorrhages in Wernicke Encephalopathy. *J. Neuroophthalmol.* **2010**, *30* (1), 54–58. https://doi.org/10.1097/WNO.0b013e3181ceb4d0.
(146) Leon, J.; Carbunar, O. Wernicke Encephalopathy Mimicking Idiopathic Intracranial Hypertension. *Neurology* **2019**, *92* (15 Supplement 1).
(147) Hwang, P.; Chalia, M. Eyes Make Brain Go Crazy. *Neurology* **2019**, *92* (15 Supplement 1).
(148) Serlin, T.; Moisseiev, E. Fundus Findings in Wernicke Encephalopathy. *Case reports in ophthalmology*, 2017, *8*, 406–409.
(149) Jalil, A.; Usmani, H. A.; Khan, M. I.; Blakely, E. L.; Taylor, R. W.; Vassallo, G.; Ashworth, J. Bilateral Paediatric Optic Neuropathy Precipitated by Vitamin B12 Deficiency and a Novel Mitochondrial DNA Mutation. *International Ophthalmology*, 2013, *33*, 687–690.
(150) Yetgin, S.; Derman, O.; Dogan, M. A Pediatric Patient with Recurrent Pseudotumor Cerebri and Vitamin B 12 Deficiency. *Pediatric Hematology and Oncology*, 2006, *23*, 39–43.
(151) Monferrer-Adsuara, C.; Garcia-Villanueva, C.; Mata-Moret, L.; Ortiz-Salvador, M.; Remoli-Sargues, L.; Cervera-Taulet, E. Case Report: Nutritional and Toxic Optic Neuropathy: A Diagnostic Dilemma. *Optom. Vis. Sci. Off. Publ. Am. Acad. Optom.* **2020**, *97* (7), 477–481.
(152) Incecik, F.; Herguner, M. O.; Karagun, B.; Altunbasak, S. Pseudotumor Cerebri Associated with Vitamin B12 Deficiency. *European Journal of General Medicine*, 2014, *11*, 121–122.

(153) Chiarello, F.; Marini, E.; Ballerini, A.; Ricca, V. Optic Neuropathy Due to Nutritional Deficiency in a Male Adolescent with Avoidant/Restrictive Food Intake Disorder: A Case Report. *Eat. Weight Disord. - Stud. Anorex. Bulim. Obes.* **2018**, *23* (4), 533–535. https://doi.org/10.1007/s40519-017-0409-6.

(154) Singanamalla, B.; Madaan, P.; Saini, L.; Sankhyan, N. Vitamin B12 Deficiency: An Association or Etiology of Pseudotumor Cerebri in An Infant. *Indian Journal of Pediatrics*, 2020, *87*, 658–659.

(155) Sethi, H. S.; Naik, M.; Gandhi, A. Megaloblastic Anemia and Bilateral Disc Edema: An Enigma... Have We Figured It out Yet? *Taiwan J. Ophthalmol.* **2020**, *10* (1), 71–75.

(156) Chu, C.; Scanlon, P. Vitamin B12 Deficiency Optic Neuropathy Detected by Asymptomatic Screening. *BMJ Case Reports*, 2011.

(157) Ata, F.; Bint I Bilal, A.; Javed, S.; Shabir Chaudhry, H.; Sharma, R.; Fatima Malik, R.; Choudry, H.; Bhaskaran Kartha, A. Optic Neuropathy as a Presenting Feature of Vitamin B-12 Deficiency: A Systematic Review of Literature and a Case Report. *Annals of Medicine and Surgery*, 2020, *60*, 316–322.

(158) Petramfar, P.; Hosseinzadeh, F.; Mohammadi, S. S. *Pseudo-Foster Kennedy Syndrome as a Rare Presentation of Vitamin B12 Deficiency*. Iranian Red Crescent Medical Journal. https://sites.kowsarpub.com/ircmj/articles/16576.html (accessed 2020-09-29). https://doi.org/10.5812/ircmj.24610.

(159) Ornek, N.; Onaran, Z.; Ornek, K.; Buyuktortop, N. Bilateral Consecutive Optic Neuropathy in a Patient with Thrombophilia. *BMJ case reports*, 2013, *2013*.

(160) Rani, U.; Imdad, A.; Beg, M. Rare Neurological Manifestation of Celiac Disease. *Case reports in gastroenterology*, 2015, *9*, 200–205.

(161) Pathmanandavel, K.; Gupta, S.; Dutt, S.; Wong, M.; Williams, A. Unusual Presentation of Coeliac Disease with Idiopathic Intracranial Hypertension. *J. Paediatr. Child Health n/a* (n/a). https://doi.org/10.1111/jpc.15182.

(162) Frohman, L. P. A Profile of Neuro-Ophthalmic Practice Around the World. *J. Neuroophthalmol.* **2018**, *38* (1), 47–51. https://doi.org/10.1097/WNO.0000000000000582.
